# Simplified Preservation of Equivalent Pathways Spectroscopy

**DOI:** 10.1021/jacsau.3c00312

**Published:** 2023-10-11

**Authors:** Evgeny Nimerovsky, Abel Cherian Varkey, Myeongkyu Kim, Stefan Becker, Loren B. Andreas

**Affiliations:** Department of NMR based Structural Biology, Max Planck Institute for Multidisciplinary Sciences, Am Fassberg 11, Göttingen 37077, Germany

**Keywords:** magic-angle spinning, nuclear magnetic resonance spectroscopy, heteronuclear dipolar recoupling, SPEPS, preservation of equivalent pathways, FLAN conditions

## Abstract

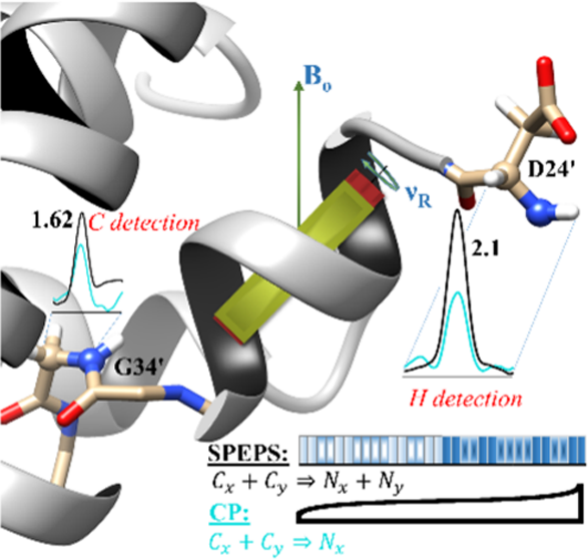

Inspired by the recently proposed transverse mixing optimal control pulses (TROP) approach for improving signal in multidimensional magic-angle spinning (MAS) NMR experiments, we present simplified preservation of equivalent pathways spectroscopy (SPEPS). It transfers both transverse components of magnetization that occur during indirect evolutions, theoretically enabling a √2 improvement in sensitivity for each such dimension. We compare SPEPS transfer with TROP and cross-polarization (CP) using membrane protein and fibril samples at MAS of 55 and 100 kHz. In three-dimensional (3D) (H)CANH spectra, SPEPS outperformed TROP and CP by factors of on average 1.16 and 1.69, respectively, for the membrane protein, but only a marginal improvement of 1.09 was observed for the fibril. These differences are discussed, making note of the longer transfer time used for CP, 14 ms, as compared with 2.9 and 3.6 ms for SPEPS and TROP, respectively. Using SPEPS for two transfers in the 3D (H)CANCO experiment resulted in an even larger benefit in signal intensity, with an average improvement of 1.82 as compared with CP. This results in multifold time savings, in particular considering the weaker peaks that are observed to benefit the most from SPEPS.

## Introduction

Heteronuclear recoupling elements are essential components of magic-angle spinning^1^ (MAS) solid-state NMR experiments. These elements connect dipolar-coupled spin pairs,^2^ allowing for the enhancement of low γ signals, and the dispersion of resonances in multidimensional spectra. Multidimensional experiments with dipolar recoupling elements at ultrafast MAS rates^3−7^ with proton detection^8−12^ are widely used for amino acid assignment and for structure determination and are applied in both materials and biological samples.^7,13−17^

The signal-to-noise ratio (SNR) of multidimensional experiments strongly depends on the efficiency of the recoupling elements. Traditionally, the cross-polarization (CP) element^18^ is used to recouple heteronuclear dipolar interactions between spin pairs under Hartmann–Hahn conditions.^19^ Initially, the element started as an experiment with constant power amplitude^20^ and was subsequently modified to include modulation (most commonly a power ramp) to improve transfer efficiency and reduce the influence of experimental imperfections.^21−39^ However, for low γ spin pairs like ^13^C–^15^N, the experimental transfer efficiency of the conventional CP element is relatively low for nondeuterated biological samples.^17,40^ It impacts the sensitivity of spectra used for amino acid assignments, especially when ^13^C–^15^N transfers are implemented twice in the same experiment.

Simultaneously transferring both components of the transverse magnetization between heteronuclear dipolar-coupled spin pairs is an elegant way to enhance SNR in multidimensional NMR experiments.^41^ This sensitivity-enhancement method, dubbed “preservation of equivalent pathways” (PEP) was proposed by Cavanagh and Rance in 1990^42^ for liquid-state NMR. The two components are acquired together, but with alternation of the sign of one component every other scan such that the real and imaginary components can be deconvoluted during data processing—a procedure known as echo/anti-echo mode acquisition.^43^ The measured signal contains contributions from both transverse components, rotated by an isotropic chemical shift interaction during the indirect dimension. In ideal cases, this provides a √2 improvement in SNR,^42,44,45^ compared to similar experiments where the amplitude of only one transverse component was recorded.

In liquid-state NMR experiments, the J-coupling interaction^46^ is usually used to transfer both transverse components between spin pairs.^42,44^ For solid-state NMR, dipolar couplings are recoupled in the conventional CP element,^19,38^ which transfers only one of the components between dipolar-coupled spin pairs. Therefore, until recently, the PEP scheme had been mainly incorporated into solid-state experiments with recoupling of homonuclear correlations,^47−49^ γ-prepared elements,^50−52^ or for static/oriented samples.^53−56^

In 2022, Blahut et al.^41^ introduced the transverse mixing optimal control pulses (TROP), which simultaneously transferred both transverse components via dipolar couplings. They showed that for deuterated samples, the gain in SNR could be more than √2 for each indirect dimension.

Inspired by their work, we have developed simplified preservation of equivalent pathways spectroscopy (SPEPS) for the simultaneous transfer of both transverse components. We demonstrate it in nondeuterated samples. The element has a simple structure, a straightforward optimization procedure, and is robust with respect to radiofrequency field (rf-field) mismatches. It is particularly efficient for resonances that appear to be negatively affected by dynamics or high-proton-density environments in the CP spectra.

## Results and Discussion

**Figure 1:**
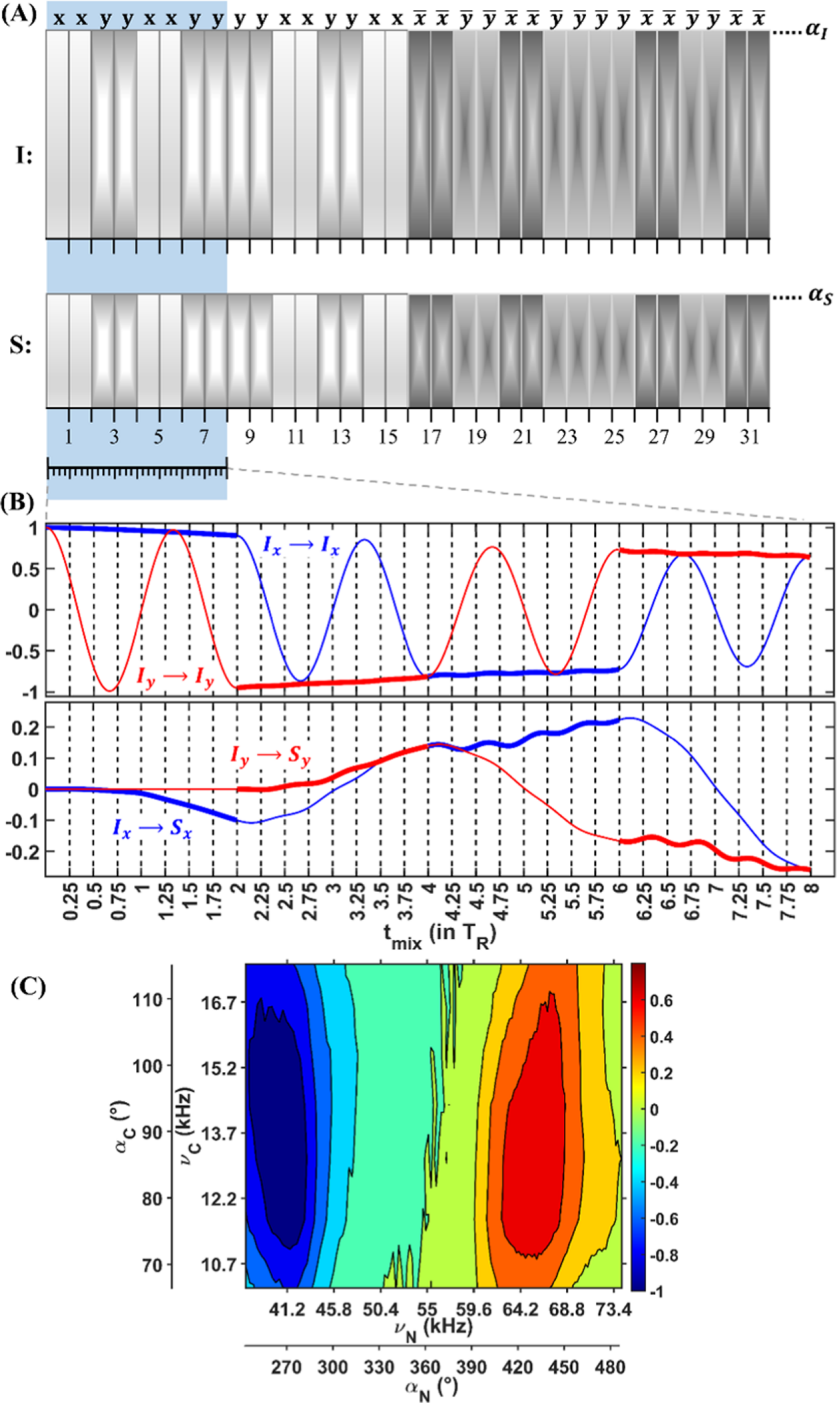
^13^CA → ^15^N SPEPS. (A) Each repeated SPEPS element consists of 32 pulses, with each pulse having the length of one rotor period (*T*_R_) and flip angles: α_I_ (α_I_ = ν_I_*T*_R_) and α_S_ (α_S_ = ν_S_*T*_R_) on I and S channels, respectively (*T*_R_ = 1/ν_R_, ν_R_ is MAS in kHz). (B) Simulated evolution of I_*x*_, I_*y*_, S_*x*_, and S_*y*_ operators during the first eight rotor periods. (C) Experimental signal intensity of the ^13^C–^15^N transfer (at *t*_mix_ = 2.91 ms) as a function of the applied rf-field strengths (flip angle values). The data were acquired on a 600 MHz spectrometer with 55 kHz MAS using the S31N M2 sample. Further details of simulations and experiments are presented in the Experimental Methods Section and in the SI.

The basic SPEPS element is depicted in Figure 1A. It contains 32 rotor-synchronized pulses, each with a length of one rotor period. The phase cycling of the pulses on both channels is identical and equals: *x*,*x*,*y*,*y*,*x*,*x*,*y*,*y*,*y*,*y*,*x*,*x*,*y*,*y*,*x*,*x*,*x̅*,*x̅*,*y̅*,*y̅*,*x̅*,*x̅*,*y̅*,*y̅*,*y̅*,*y̅*,*x̅*,*x̅*,*y̅*,*y̅*,*x̅*,*x̅* (XY-16 based phase cycling^57^). The basic element can be extended in length by repetition, allowing facile application over a range of spinning frequencies. The flip angle values of each pulse on the I and S channels are α_I_ (α_I_*=* ν_I_*T*_R_) and α_S_ (α_S_ = ν_S_*T*_R_), respectively (*T*_R_ = 1/ν_R_, ν_R_ is MAS in kHz). The optimal flip angles of the pulses are the flip angles that fulfill the FLAN conditions^58^ (briefly explained in the Supporting Information (SI)) and Hartmann–Hahn conditions^19^ simultaneously (Figure S1 in the SI). For example, possible optimal rf-field strengths (in kHz) for double-quantum (DQ) and zero-quantum (ZQ) transfers are (0.25, 0.75 ν_R_) and (0.25, 1.25 ν_R_), respectively. Herein, we focus primarily on the DQ condition, which has a low rf-field requirement for both rf channels.

Figure 1B shows simulations that explain the mechanism of simultaneous transfer of both transverse components, showing evolutions of transverse (I_*x*_, I_*y*_) and (S_*x*_, S_*y*_) operators during the first eight rotor periods. When the initial operator, I*_j_* (*j* = *x* or *y*), has the same phase as the applied rf-field pulses, the operator becomes locked and the transfer from I*_j_* to S*_j_* occurs (indicated by thick lines). When the initial operator and the applied pulse phase differ by 90°, the I*_j_* operator is inverted after two rotor periods have elapsed (indicated by thin lines). This construction enables the simultaneous transfer of I_*x*_ → S_*x*_ and I_*y*_ → S_*y*_ operators within the SPEPS pulse. It also results in a slower transfer of magnetization. However, the experimental optimum transfer time for ramped CP is also long such that the optimal transfer time for SPEPS ends up shorter than that for ramped CP (vide infra).

A successful pulse sequence must be robust against rf-field mismatches or inhomogeneity and should remain effective for a range of spin systems. Fortunately, the requirement for complete inversion of the nontransferred spins is relaxed when the SPEPS element is implemented with the full, extended, phase cycling scheme, as shown in Figures S2 and S3 in the SI. Figure S4 shows that there is a small dependence of the SPEPS element on the number of proximate proton spins and on the rf-field inhomogeneity.

Experimental data confirm the performance of SPEPS. From the experimental SPEPS profile (Figure 1C), we observe two regions of optimal RF-field strengths on the I and S channels for DQ (negative) and ZQ (positive) transitions: 0.24, 0.75 and 0.24, 1.21 ν_R_. Our focus was on SPECIFIC CP^22,59^ such that we did not consider higher rf-field on the ^13^C channel. The DQ condition also appears to be more efficient than the ZQ condition (Figure S5 in the SI).

**Figure 2:**
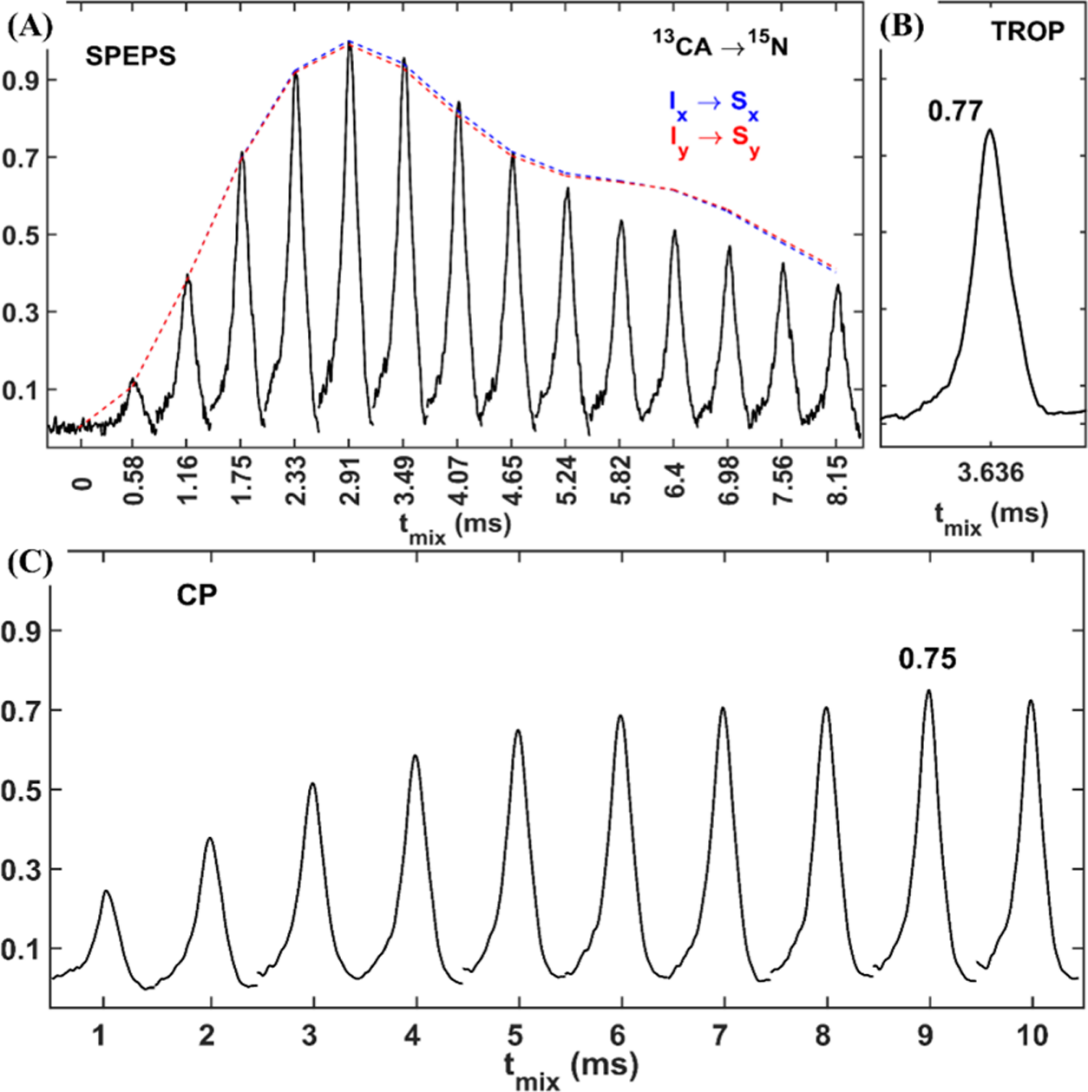
^13^CA → ^15^N buildup curves, normalized to the SPEPS maximum intensity. (A) Simulated SPEPS (I_*x*_ → S_*x*_ blue and I_*y*_ → S_*y*_ red) and experimental (average I_*x*_ → S_*x*_ and I_*y*_ → S_*y*_ black spectra) transfer efficiency plotted against mixing time. In the simulations, a three-spin system (I_2_S) was used with heteronuclear and homonuclear dipolar coupling values of 1.1 and 2.5 kHz, respectively. Pulses of 284° (43.4 kHz) and 79° (12.1 kHz) were applied on the I and S channels, respectively. (B) Experimental TROP spectrum at a fixed 3.636 ms mixing time for 55 kHz MAS. (C) Experimental CP transfer efficiency plotted against mixing time. In all figures, all experimental spectra have been normalized using the maximal intensity obtained from the SPEPS experiments. The data were acquired on a 600 MHz spectrometer with 55 kHz MAS and the S31N M2 sample. In the experiments, the global SPEPS phase cycling was shifted by 90° from scan to scan to balance the *x*- and *y*-component transfer efficiency. Further details of simulations and experiments are presented in the Experimental Methods Section and in the SI.

Figure 2 shows SPEPS (Figure 2A) and CP (Figure 2C) signal intensity as a function of mixing time as well as the TROP spectrum (Figure 2B). TROP is a transfer element designed with a fixed mixing time, while SPEPS and CP elements can be optimized with respect to time. The behaviors of the experimental and simulated SPEPS curves under the DQ conditions are in good agreement with each other (Figure 2A). In simulations (dashed lines, normalized to the maximum simulated intensity), a transfer efficiency of approximately 46% is observed as compared to the simulated 90° excitation. The same efficiency is simulated for each transverse component (I_*x*_ → S_*x*_ blue curves and I_*y*_ → S_*y*_ red curves). Experimentally, we estimated an efficiency of approximately 31% by comparison with a direct polarization (a 90° excitation; Figure S6 in the SI). For CP (Figure 2C), longer mixing times are required to reach maximal efficiency (9 ms) compared to that of SPEPS (2.91 ms). For this nondeuterated sample, SPEPS exhibits higher ^13^C–^15^N transfer efficiency compared to TROP and CP (SPEPS/TROP/CP – 1:0.77:0.75). In Figures S7–S8, we show the experimental optimization profiles for CP (Figure S7) and TROP (Figure S8), demonstrating that conditions were carefully adjusted to maximize the transfer in each case.

It is worth mentioning that in all experiments traditional ramped CP elements were used for ^1^H–^13^C and ^1^H–^15^N transfers. As investigated by different research groups,^34,60−68^ the ramped CP elements result in a volume- and orientation-selective transfer due to rf-field inhomogeneity. The SPEPS element itself is also affected by rf-field inhomogeneity (Figure S4). We would therefore recommend to optimize all transfer elements of SPEPS-based sequences, rather than taking the optimization from the CP-only sequences. We did this for the data acquired at the 850 spectrometer, but not for the 600 MHz data, where the values from CP-only-based transfer were taken directly for the SPEPS-based sequences. The difference amounted to 5 to 10% at the 850 MHz instrument.

Figure S9 shows one-dimensional (1D) comparisons of SPEPS, TROP, and CP showing improved performance for SPEPS for ^13^C–^15^N transfer compared to CP and TROP at an 850 MHz spectrometer using 55 kHz MAS (Figure S9A) and a similar transfer efficiency compared to CP at a 950 MHz spectrometer using 100 kHz MAS (Figure S9B). By contrast, CP remained the most efficient for ^15^N–^1^H transfer (Figure S10). Nominally, only a single transverse component is transferred in all of these 1D experiments.

At 55 kHz and 850 MHz (Figure S9A), SPEPS exhibits higher ^13^C–^15^N transfer efficiency compared to TROP and CP (SPEPS/TROP/CP – 1:0.81:0.85). Similar 1D signals were seen for SPEPS and CP at 100 kHz (Figure S9B), indicating that SPEPS should outperform CP in multidimensional spectra. The TROP element was not investigated at 100 kHz MAS due to the limitations of the rf-field powers of the probe, and since a suitable TROP pulse has not been reported for 100 kHz MAS.

In Figure S11, we present comparisons of 2D SPEPS, TROP, and CP spectra with nitrogen and carbon detection. Here, we expect a √2 gain in SNR for SPEPS and TROP elements as compared with the 1D signal of Figure S9 since SPEPS and TROP transfer both transverse components of magnetization. For ^13^C → ^15^N transfers (Figure S11A), SPEPS is notably more efficient than the TROP element for residues with a high-proton-density environment^69^ (e.g., glycine and proline residues). For ^15^N → ^13^C transfers (Figure S11C), SPEPS demonstrates much higher transfer efficiency compared to TROP and CP. This is unsurprising for the TROP element since it was specifically developed for ^13^C → ^15^N transfer.^69^

The ^13^C–^15^N SPEPS element can be incorporated into the proton-detected three-dimensional (3D) (H)CANH experiment, where ^1^H–^13^C and ^15^N–^1^H transfers are implemented with conventional CP elements. The data requires an echo/anti-echo-like manipulation in the time domain before Fourier transformation, as previously described.^70^ A script for this purpose is provided in the SI as a .txt file and is combined with optional drift correction.^71^

**Figure 3:**
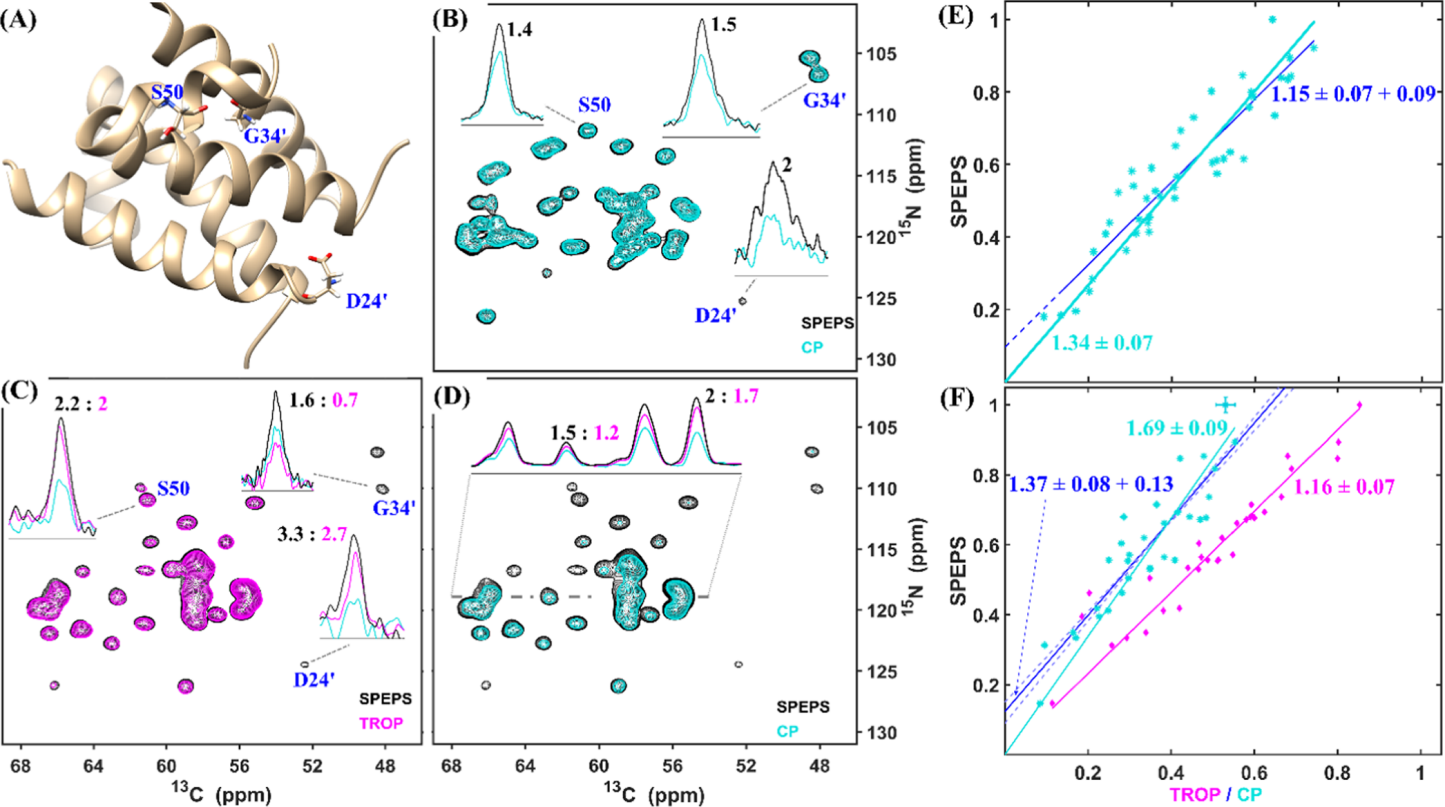
Comparison of 3D (H)CANH spectra recorded with SPEPS, TROP, and CP for the M2 protein from Influenza A. (A) Structure of influenza A M2 from PDB 2N70.^72^ (B, E), 950 MHz data at 100 kHz MAS recorded for WT M2. (C, D, F), 850 MHz data at 55 kHz MAS recorded for S31N M2. (B–D) ^13^C–^15^N projections from 3D spectra obtained with SPEPS (black), TROP (magenta), and CP (cyan) elements for ^13^C–^15^N transfers. (E, F) Comparison of the peak intensities for selected residues: magenta – SPEPS (axis *y*) and TROP (axis *x*); cyan – SPEPS (axis *y*) and CP (axis *x*). The lines were obtained with linear least-squares fitting either with (cyan, magenta) or without (blue) fixing the intercept to zero. For (F), three pairs of residues were scaled down by a factor of 2 due to chemical shift degeneracy. Six Leucine residues were not included in the comparison due to signal overlap. The errors in the slopes were obtained by calculating the standard deviation values. The dashed lines represent the fitting lines with fixed slope values (from solid blue lines) ± errors. Further details of simulations and experiments are presented in the Experimental Methods Section and in the SI.

Figure 3 displays the corresponding data recorded from the 3D (H)CANH experiments. The tetrameric arrangement of membrane protein influenza A M2 is shown in Figure 3A, with residues D24, G34, and S50 indicated. In Figure 3B–D, we present a comparison of ^13^C–^15^N projections from 3D (H)CANH spectra obtained using SPEPS (black), TROP (magenta), and CP (cyan) elements, acquired on a 950 MHz spectrometer, with 100 kHz MAS (B) and at 850 MHz, with 55 kHz MAS (C–D). Figure 3E,F compares all detected peak intensities from the SPEPS spectrum with the corresponding peaks from the CP (blue)- and TROP (magenta)-based spectra. Cyan and magenta lines in Figure 3E,F are linear fits with the y-intercept fixed to 0 and show the average improvement in peak intensity. The *y*-intercepts of the blue trend lines (linear fits of slope and intercept) indicate that the benefit of SPEPS is proportionally larger for weaker peaks. Note that the slope in this case does not indicate the improvement, and in fact, a slope of zero could be ideal, if all peaks in the SPEPS spectrum would be equally intense. The dashed lines define the range of possible *y*-intercept values, resulting after refitting the data with the slope plus, or minus, the error in the slope (dashed lines). Both dashed lines have positive *y*-intercepts, confirming the aforementioned conclusion. All errors are reported at 1 standard deviation.

**Figure 4:**
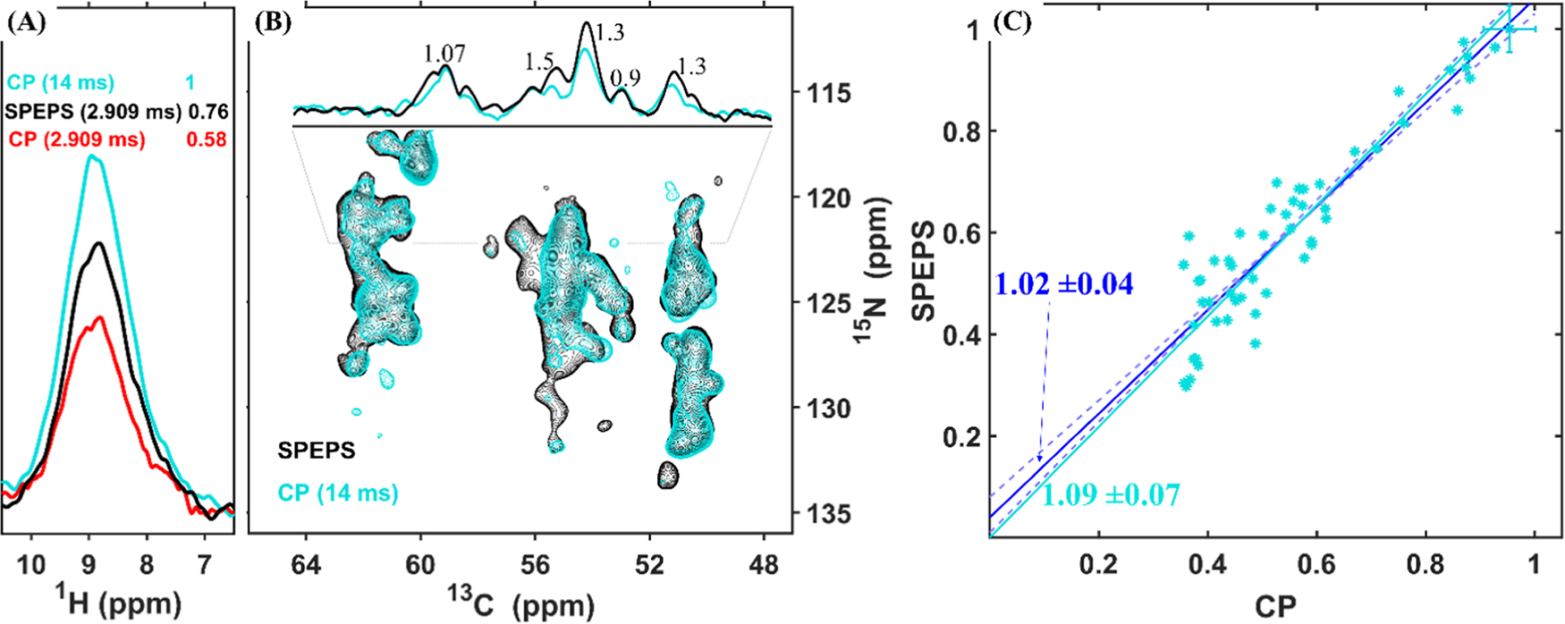
Comparison of 3D (H)CANH spectra recorded with SPEPS and CP for the α-Synuclein fibril. (A) 1D (HCAN)H spectra with CP (2.909 ms, red and 14 ms, cyan) and SPEPS (2.909, black) elements. (B) ^13^C–^15^N projections from 3D spectra obtained with SPEPS (black) and CP (14 ms, cyan) elements for ^13^C–^15^N transfers. (C) Comparison of peak intensities for all peaks detected above the noise threshold of the CP experiment: SPEPS (*y*-axis) and CP (*x*-axis). The lines were obtained with linear least-squares fitting either with (cyan) or without (blue) fixing the intercept to zero. The data were acquired using an 850 MHz spectrometer with 55 kHz MAS. Further details of simulations and experiments are presented in the Experimental Methods Section and in SI.

The observation that SPEPS improves the signals of weaker peaks in the M2 spectra and the fact that SPEPS requires shorter transfer times than CP suggest that the benefit of SPEPS can be expected to be sample dependent. We therefore selected a fibrillar α-Synuclein sample and assessed the performance of SPEPS on an 850 MHz spectrometer (Figure 4) in comparison with CP. In Figure 4A, a comparison of 1D (HCAN)H spectra reveals a 1.3-fold higher signal using CP (14 ms, cyan) vs SPEPS (black). Notably, the CP optimum, at 14 ms, is longer than the optimum 9 ms for M2 (Figure 2), which is consistent with longer relaxation times and more ideal performance of CP. Consistent with the M2 data, at a shorter CP mixing time (red) that matches the optimum for SPEPS, a significantly lower signal is observed. Figure 4B presents a comparison of ^13^C–^15^N projections from a 3D (H)CANH experiment with SPEPS (black) and CP (14 ms mixing time, cyan) elements. Figure 4C compares all detected peak intensities from the CP spectrum to the corresponding peaks from the SPEPS-based spectrum. The additional √2 gain in SNR for the SPEPS element, obtained from acquiring the 3D (H)CANH experiment, results in the expected slight improvement over CP by a factor of 1.09 (Figure 4C).

It is worth noting that theoretical simulations that do not consider relaxation would predict a higher transfer efficiency for CP than for SPEPS. Adiabatic CP, with an efficiency up to 100%,^39,73,74^ exceeds the simulated transfer efficiency of the SPEPS element, which suggests that for samples with very long relaxation times, SPEPS may not outperform CP. It appears that the fibrillar sample comes closer to this situation. Nevertheless, a slight improvement was observed with SPEPS, suggesting that it can be applied across different sample types (Table 1).

**Table 1:** Average SNR Improvement from the Three Samples^a^

sample	M2 WT	M2 S31N	αSYN
^1^H Larmor frequency	950 MHz	850 MHz	850 MHz
MAS	100 kHz	55 kHz	55 kHz
SPEPS vs CP	1.34	1.69	1.09
SPEPS vs TROP		1.16	

a Summary of SNR improvement factors obtained from peak intensities of 3D (H)CANH spectra with SPEPS vs TROP or CP elements (Figures 3 and 4).

The 3D (H)CANCO spectrum is a more challenging case since it requires two magnetization transfers between carbon and nitrogen spins. This experiment can be crucial for site-specific assignment, as it links the three sequential backbone resonances.^75,76^ However, the 3D (H)CANCO experiment is hindered by a characteristically low signal-to-noise ratio (SNR) and typically is recorded with large sample volumes or long experimental times.

**Figure 5:**
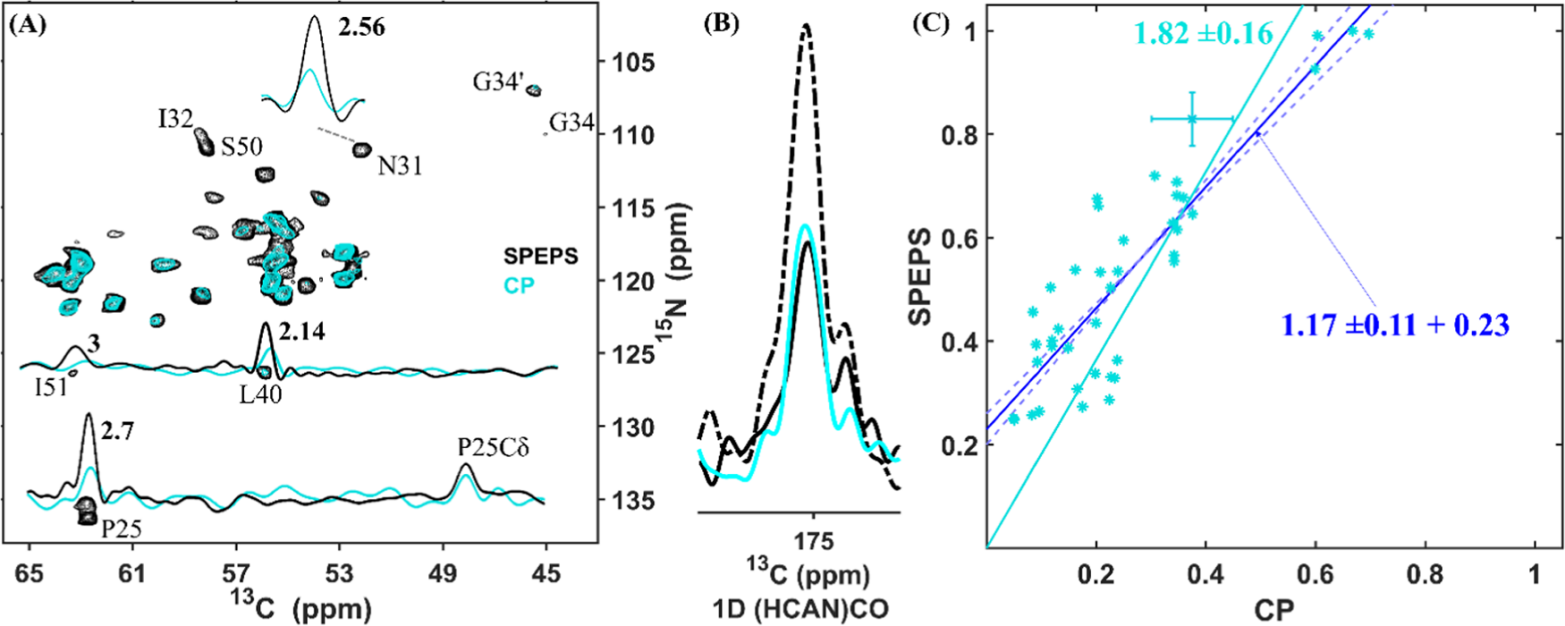
3D (H)CANCO spectra of S31N M2 acquired with either SPEPS (black) or CP (cyan). (A) ^13^C–^15^N projections for spectra acquired with SPEPS (black) or CP (cyan) for ^13^C–^15^N transfers. (B) 1D (HCAN)CO spectra with SPEPS (solid black, only one component; dash-dot black, the sum of both components) and CP (cyan) elements. (C) Comparison of peak intensities for all peaks detected above the noise threshold: SPEPS (*y*-axis) and CP (*x*-axis). The lines were obtained with linear least-squares fitting either with (cyan) or without (blue) fixing the intercept to zero. The data were acquired using a 600 MHz spectrometer with 55 kHz MAS. The same ^1^H–^13^CA CP conditions were applied for both experiments. Further details of simulations and experiments are presented in the Experimental Methods Section and in the SI.

The SPEPS element provides a practical solution to enhance the performance of the 3D (H)CANCO experiment. We observed an improvement in SNR by a factor of almost 2, as shown in Figure 5. From the 1D comparison, we observe a similar intensity for the SPEPS and CP elements (Figure 5B), when only one component is detected for the SPEPS (solid black). The sum of the signals from both components (dash-dot black) provides the expected 2-fold improvement in SNR. Figure 5A compares the CA-N projections from the 3D (H)CANCO experiments obtained with the SPEPS (black) and CP (cyan) elements. Figure 5C presents a comparison of the peak intensities using the SPEPS (*y*-axis) and CP (*x*-axis) elements. On average, SPEPS provides a 1.82-fold SNR improvement factor, slightly below the expectation of 2 based on the 1D comparison.

Also here, the *y*-intercept of 0.23 of the dark blue trend line in Figure 5C indicates that the benefit of SPEPS is proportionally larger for weaker peaks. This facilitates the interpretation of the spectrum since the weaker peaks become less likely to be obscured by overlapping signals. Furthermore, the required acquisition time is determined by the intensity of the weakest peak of interest. Since the required time scales with the square of the SNR, the observed improvement translates into a multiple-fold reduction in the required spectrometer time.

## Conclusions

In summary, this work introduces the SPEPS transfer element, which improves SNR by transferring both components of magnetization that evolve in indirect dimensions. The SPEPS element has a simple structure and a facile optimized procedure and exhibits robust performance in the presence of rf-field inhomogeneity. We compared the transfer efficiency of SPEPS with the conventional CP element^38^ and the recently proposed TROP element^41^ and found a substantial improvement. A more impressive enhancement was seen for the 3D (H)CANCO experiment that contains two SPEPS transfers, where we observed an average SNR improvement factor of 1.82 compared to CP. The short transfer time of SPEPS is expected to facilitate measurements of peaks affected by short relaxation times during the transfer, such as may occur for residues with a high-proton-density environment or for nonideal membrane protein preparations. Indeed, a particular benefit was observed for the more challenging, low-intensity peaks, ultimately facilitating the NMR-based study of complex biomolecular systems with higher fidelity.

## Experimental Methods

### Simulations

SPEPS simulations were performed using in-house MATLAB scripts to compute the numerical solution of the equation of motion.^77^

For simulations in Figure 1B, we used a two-spin system (representing CA and N spins) with 11 kHz dipolar coupling. For simulations in Figure 2A, a three-spin system (representing CO, CA, and N spins) was used with heteronuclear (CA-N) and homonuclear (CO-CA) dipolar coupling values of 1.1 and 2.5 kHz, respectively, and with the following values for [chemical shift offset; chemical shift anysotropy] in kHz: [−0.6;9] (N); [−1.7;8] (CA); and [15;17] (CO). Pulses of 284° (43.4 kHz) and 79° (12.1 kHz) were applied on the *N* and *C* channels, respectively, slightly away from the expectation of 270 and 90° such that each pulse produces a net 180° rotation over two rotor periods.

### Sample Preparation

#### Influenza A M2 Samples

Influenza A wild-type M2 and S31N M2 proteins (residues 18–60) were prepared according to the protocols in the references.^78,79^ The samples were packed into either Bruker 1.3 or 0.7 mm rotors via centrifugation.

#### α-Synuclein Sample

α-Synuclein was recombinantly produced in *Escherichia coli* strain BL21(DE3) as previously described.^80^^13^C,^15^N-labeled samples were expressed in a minimal medium supplemented with ^15^NH_4_Cl and ^13^C_6_-d-glucose (Cambridge Isotope Laboratories and Sigma-Aldrich). The protein was finally dialyzed against buffer (50 mM HEPES, 100 mM NaCl, pH 7.4) to obtain a 0.3 mM solution, and the resulting solution was stored at −80 °C until use.

Vesicles were prepared from lipid films. A chloroform mixture of 1-palmitoyl-2-oleoyl-glycero-3-phosphocholine (POPC), 1-palmitoyl-2-oleoyl-*sn*-glycero-3-phosphate (POPA) was placed under a N_2_-stream to form lipid films, and the film was lyophilized overnight. Buffer (50 mM HEPES, 100 mM NaCl, pH 7.4) was added, and SUVs were obtained by repeated sonication to produce a solution of 1.5 mM POPC and 1.5 mM POPA.

Monomeric α-synuclein (in buffer containing 50 mM Hepes, 100 mM NaCl, and pH 7.4) was centrifuged for 1 h at 55,000 rpm using a TLA-100.3 rotor in an Optima MAX-XP tabletop ultracentrifuge (both Beckman Coulter) at 4 °C. The supernatant was added to a solution of phospholipid small unilamellar vesicles (SUVs) and NaN_3_ (0.02 wt %) to obtain a final protein concentration of 70 μM and a molar lipid-to-protein ratio of 10 in buffer (50 mM HEPES, 100 mM NaCl, pH 7.4) and continuously shaken with 1% (molar ratio) preformed fibril seeds at 180 rpm in a Multitron incubator (Infors HT, Bottmingen, CH). The seeds were obtained by sonicating preformed a-Syn fibrils that were prepared previously.^81^ After 96 h, the fibrils were harvested by centrifuging at 152,460*g* (TLA-100.3 rotor in an OptimaTM MAX-TL) for 1 h at 4 °C. The supernatant was removed, and the resulting pellet was washed with fresh buffer and centrifuged again at 212,940*g* for 10 min at 18 °C. Excess moisture was removed, and the pellet was immediately packed into a 1.3 mm rotor.

### Solid-State NMR Spectroscopy

#### Optimizing SPEPS Experiments

^13^C and ^15^N 90° hard pulses were precisely calibrated (crucial for such experiments, where the pair of 90° pulses preceding the SPEPS transfer inverts the transverse component in alternating increments). The initial rf-field power values of the SPEPS element were set according to hard pulse calibrations and optimized empirically around these values. For ^13^C and ^15^N channels, the optimal rf-field values correspond to approximately 0.25 and 0.75 ν_R_, respectively. The carrier frequencies of ^13^CA and ^15^N were set to 53.7 and 116 ppm, respectively.

#### 600 MHz

1D (HCAN)H (Figures 1C and 2) and 3D (H)CANCO (Figure 5) experiments were acquired on a Bruker Avance III HD spectrometer operating at 14.1 T (600 MHz ^1^H frequency) using a DVT600W2 BL1.3 mm HXY probe. The experiments were performed at 55 kHz MAS, and the temperature of the nitrogen cooling gas was set to 245 K with 1000 to 1300 L/h.

#### 850 MHz

3D (H)CANH experiments (Figures 3C–D and 4) were acquired on an Avance III spectrometer operating at 19.97 T (850 MHz ^1^H field strength), equipped with a 1.3 mm HCN MAS probe at 55 kHz MAS. The temperature of the nitrogen cooling gas was set to 245 K.

#### 950 MHz

3D (H)CANH experiments (Figure 3B) were acquired on a Bruker Avance III HD spectrometer operating at 22.3 T (950 MHz ^1^H frequency), equipped with a 0.7 mm HCDN MAS probe at 100 kHz MAS. The temperature of the nitrogen cooling gas was set to 252 K with 550 L/h.

In all experiments, the estimated sample temperature (accounting for frictional heating from the MAS) was 288–298 K. For decoupling of the heteronuclear dipolar interactions, SW_f_-TPPM^82^ was used on the proton channel and WALTZ-16^83^ was used on heteronuclear channels. MISSISSIPPI^84^ water suppression was applied for proton-detected experiments.

## Supporting Information Available

Linear drift compensation and SPEPS processing for Topspin 4 (TXT)
Linear drift compensation and SPEPS processing for Topspin 3 (TXT)
Numerical simulations of SPEPS, additional experimental data using SPEPS, TROP, and CP elements for dipolar recoupling, experimental parameters, and Bruker Topspin pulse programs implementing the SPEPS-based sequences (PDF)
