# Supplementary material for: Simplified Preservation of Equivalent Pathways Spectroscopy
Source: JACS Au. 2023 Oct 11;3(10):2763–71. doi: 10.1021/jacsau.3c00312 (PMC10598565; doi:10.1021/jacsau.3c00312)
Supplement: Supplementary file 3 — au3c00312_si_003.pdf [file au3c00312_si_003.pdf]

Simplified Preservation of Equivalent Pathways Spectroscopy

Authors: *Evgeny Nimerovsky*, Abel Cherian Varkey, Myeongkyu Kim, Stefan Becker & Loren B. Andreas**

Affiliations:

Department of NMR based Structural Biology, Max Planck Institute for Multidisciplinary Sciences, Am Fassberg 11, Göttingen, Germany

*Corresponding authors: land@mpinat.mpg.de ORCID: 0000-0003-3216-9065 and evni@mpinat.mpg.de ORCID: 0000-0003-3002-0718.

Contents

RAW DATA STATEMENT 2
A BRIEF DESCRIPTION OF FLAN CONDITIONS 2
SIMULATIONS AND EXPERIMENTS 4
EXPERIMENTAL METHODS 16
  Solid state NMR spectroscopy 16
  Compiling script (Echo/ Anti-Echo mode into STATES mode). 28
BRUKER PULSE PROGRAMS 29
  The SPEPS SHAPE file 29
  2D (H)NC 30
  2D (H)CAN 32
  3D (H)CANH 36
  3D (H)CANCO 41
REFERENCES 45

RAW DATA STATEMENT

The raw data from the experiments have been uploaded to the Zenodo open research data repository (https://zenodo.org/record/8248311) under the license: 'Creative Commons Attribution 4.0 International'. Note that the uploaded topspin codes do not contain any protection against long acquisitions that occur if this parameter is mistakenly set too low. Use only at your own risk.

A BRIEF DESCRIPTION OF FLAN CONDITIONS

Under Hartmann-Hahn conditions,[1] the applied pulses on different channels are considered as continuous spin-lock pulses. CP transfers between a pair of spins occur, when the sum (for double-quantum transfers, DQ) or difference (for zero-quantum transfers, ZQ) between rf-field strengths of these spin-lock pulses matches the rotor frequency or twice the rotor frequency:

$$\text{ZQ: } \nu_I - \nu_S = n_{ZQ}\nu_R \qquad \text{DQ: } \nu_I + \nu_S = n_{DQ}\nu_R. \qquad \text{Eqn. (S1)}$$

Here, ν_I and ν_S are applied rf-field strengths on I and S spins, respectively; ν_R is a MAS rate and n_{ZQ}, n_{DQ}=±1,±2.

Recently, we investigated the windowed CP pulse sequence (wCP), which consisted of a train of pulses, with one pulse per rotor, thus resulting also in one window every rotor period.[2] In that case, DQ and ZQ transfers were defined with the sum or difference of the flip angles resulting from each pulse:

$$\Sigma_{IS} = \alpha_I + \alpha_S = \pi + 2\pi n \text{ and } \Delta_{IS} = \alpha_I - \alpha_S = 2\pi + 2\pi n', \quad n \geq n', \qquad \text{Eqn. (S2)}$$

where $\alpha_I = 2\pi\nu_I t_I$ and $\alpha_S = 2\pi\nu_S t_S$; t_I and t_S are the lengths of each pulse on I and S channels, respectively. We demonstrated the similarity between CP and wCP sequences by using the same

description of ZQ and DQ transfers, respecting the FLAN conditions, and preserving the same properties for both sequences. These investigations provided us with a clue for developing the SPEPS element: the flip angle of the applied pulses had to be close to a FLAN condition, while changing the phase only every two rotor periods would result in either spin locking or inversion on x and y channels. We then testing different xy phase cycles.

Figure S1A displays the schematic presentation of FLAN conditions, where the circles represent the double-quantum conditions and the stars represent zero-quantum conditions. The lines represent Hartmann-Hahn conditions. The overlapping FLAN and Hartmann-Hahn conditions define the optimal flip-angle for SPEPS.

Figure S1B shows simulated build-up curves with SPEPS (BI, continuous pulses) and windowed SPEPS (BII, delay and a single pulse per one rotor period). The colorful numbers indicate the flip angle set used in (BI) and (BII). By implementing a windowed SPEPS element (window + pulse per rotor period on both channels), additional transfer conditions appear (Figure S1BII): build-up curves 5 (magenta) and 6 (cyan).

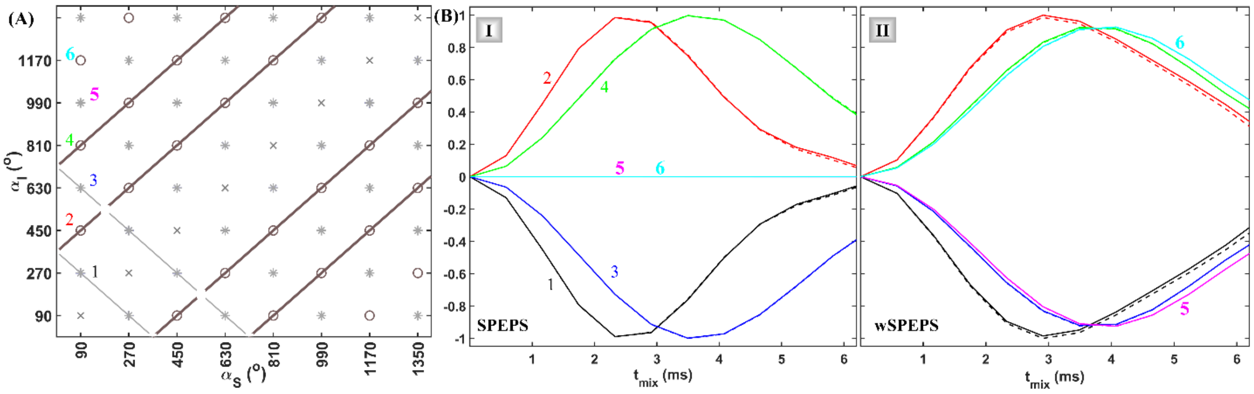

Figure S1 FLAN conditions and simulated buildup curves for heteronuclear transfer using SPEPS and windowed SPEPS (wSPEPS). (A) Flip angle values (FLAN conditions) for which the FOH was calculated (more details in Ref.[2]). Circle and stars represent positive and negative conditions. Thick and thin lines correspond to the flip angles

for CP, where the pulse length is one rotor period on both channels, and the sum or difference between rf-field values match Hartmann-Hahn conditions.[3] The diagonal (crosses) represents a special case, where positive transfer is observed if $t_I \neq t_S$.[2] The colorful numbers indicate the flip angle set used in (BI) and (BII). (BI) SPEPS buildup curves with continuous pulses. (BII) windowed SPEPS curves with half rotor period pulses every rotor period (on both channels). The rf-field strengths are doubled, compared to (BI) to maintain the same flip angle values. In (B), a three spin system (I_2S) was simulated with 1 kHz heteronuclear, 2.5 kHz homonuclear dipolar coupling values and 55 kHz MAS. The phase cycling is: $x, x, y, y, x, x, y, y, y, y, x, x, y, y, x, x, \bar{x}, \bar{x}, \bar{y}, \bar{y}, \bar{x}, \bar{x}, \bar{y}, \bar{y}, \bar{y}, \bar{y}, \bar{x}, \bar{x}, \bar{y}, \bar{y}, \bar{x}, \bar{x}$ (XY16 based phase cycling[4]).

SIMULATIONS AND EXPERIMENTS

The phase cycling scheme has a fundamental influence on the SPEPS transfer efficiency. The figure below shows the simulated dependence of SPEPS curves on XY4 (A), XY8 (B), XY16 (C) and XY32 (D) phase cycling schemes as well as flip angle values. In these simulations, we considered a two-spin system with dipolar interaction and an rf-field Hamiltonian only. The sum of the selected flip angle pairs always fulfilled the ZQ condition ($\alpha_C + \alpha_N = 180°$).

The longest phase cycling (D, XY32) provides the highest transfer efficiency, has the smallest dependence on the change of flip angle values and provides similar transfer efficiencies for $C_x \rightarrow N_x$ and $C_y \rightarrow N_y$ paths. Interestingly, for XY4 and XY32 phase cycling schemes the maximum transfer efficiency is achieved with $\alpha_C = 61°$ and $\alpha_N = 299°$ flip angle values (green curves).

The magenta curves demonstrate that achieving proper inversions of non-transferred spin operators is not a stringent requirement. In this case, the applied pulses have a 100° flip angle per two rotor periods, whereas for a proper inversion, the requirement is a 180° flip angle per two rotor periods. Interestingly, for XY16 and XY32 phase cycling schemes we observe better

transfer efficiency compared to conditions where the pulses have a 180° flip angle per two rotor periods (red curves).

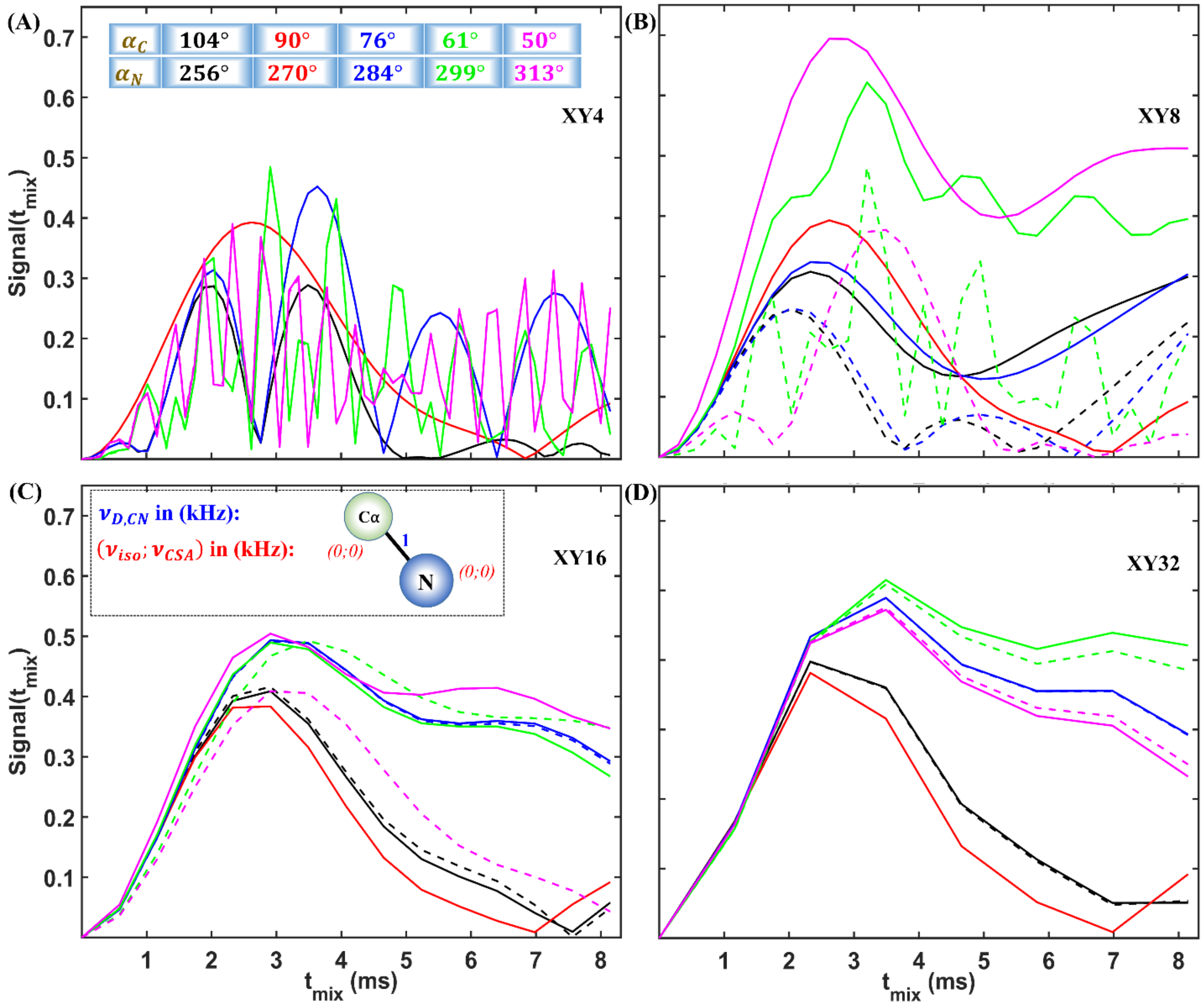

Figure S2 Two-spin system simulations were conducted to investigate the numerical dependencies of SPEPS curves on phase cycling schemes and flip angle values on carbon and nitrogen spins. The following flip angle pairs were considered: 104° and 256° - black; 90° and 270° - red; 76° and 284° - blue; 61° and 299° - green and 50° and 313° - magenta. The two-spin system was simulated with 55 kHz MAS, 1 kHz dipolar coupling value and zero values for isotropic chemical shift and chemical shift anisotropy. The applied phase cycling scheme for SPEPS elements: (A) XY4 phase cycling – x, x, y, y; (B) XY8 phase cycling - $x, x, y, y,\ x, x, y, y,\ y, y, x, x,\ y, y, x, x$; (C) XY16 phase cycling -$x, x, y, y,\ x, x, y, y,\ y, y, x, x,\ y, y, x, x,\ \bar{x}, \bar{x}, \bar{y}, \bar{y},\ \bar{x}, \bar{x}, \bar{y}, \bar{y}\ , \bar{y}, \bar{y}, \bar{x}, \bar{x},\ \bar{y}, \bar{y}, \bar{x}, \bar{x}$ and (D) XY32 phase

cycling - $x,x,y,y,\ x,x,y,y,\ y,y,x,x,\ y,y,x,x,\ \bar{x},\bar{x},\bar{y},\bar{y},\ \bar{x},\bar{x},\bar{y},\bar{y},\ \bar{y},\bar{y},\bar{x},\bar{x},\ \bar{y},\bar{y},\bar{x},\bar{x},\ y,y,\bar{x},\bar{x},\ y,y,\bar{x},\bar{x},$ $\bar{x},\bar{x},y,y,\ \bar{x},\bar{x},y,y,\ \bar{y},\bar{y},x,x,\ \bar{y},\bar{y},x,x,\ x,x,\bar{y},\bar{y},\ x,x,\bar{y},\bar{y}.$

Since our experiments involve investigating larger spin systems including proton spins, the simulated six-spin system provides a more realistic representation of the dependence of the SPEPS element on the phase cycling scheme (Figure S3). The SPEPS element with XY16 (C) and XY32 (D) exhibits similar behavior and transfer efficiency, with less sensitivity to flip angle deviations. In both phase cycling schemes, the maximum transfer efficiency is achieved at flip angles of 76° ($0.21\nu_R$) and 284° ($0.79\nu_R$) (blue curves).

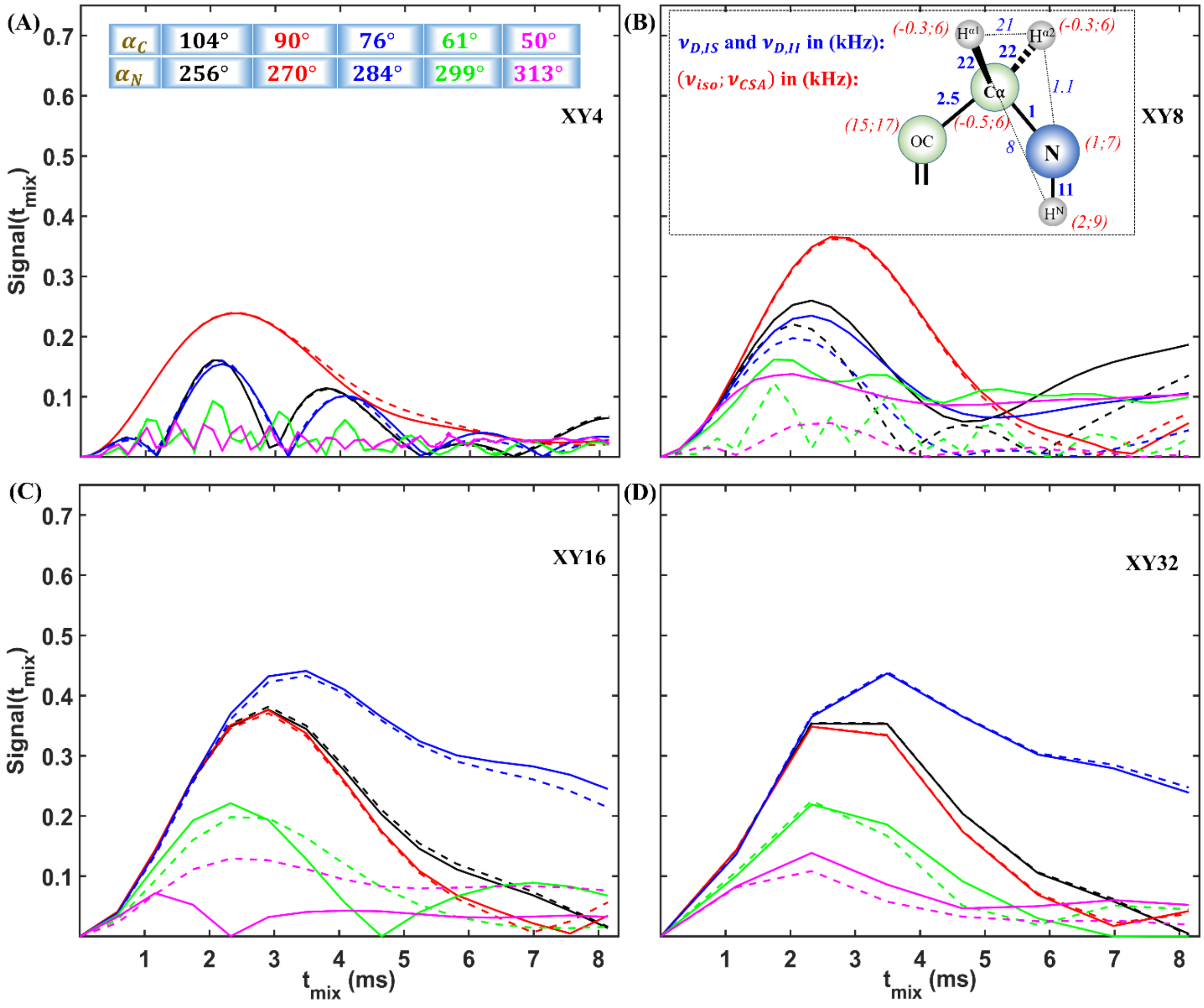

Figure S3 Six-spin system simulations were conducted to investigate the numerical dependencies of SPEPS curves on phase cycling schemes and flip angle values on carbon and nitrogen spins. The following flip angle pairs were considered: 104° and 256° - black; 90° and 270° - red; 76° and 284° - blue; 61° and 299° - green and 50° and 313° - magenta. Six spin system was simulated with 55 kHz MAS. The dipolar couplin values (ν_D), the isotropic chemical shift and and chemical shift anisotropy ($\nu_{iso}; \nu_{CSA}$) used in simulations are depicted in Figure B. The applied phase cycling scheme for SPEPS elements: (A) XY4 phase cycling – x, x, y, y; (B) XY8 phase cycling - x, x, y, y, x, x, y, y, y, y, x, x, y, y, x, x; (C) XY16 phase cycling -x, x, y, y, x, x, y, y, y, y, x, x, y, y, x, x, $\bar{x}, \bar{x}, \bar{y}, \bar{y}$, $\bar{x}, \bar{x}, \bar{y}, \bar{y}$, $\bar{y}, \bar{y}, \bar{x}, \bar{x}$, $\bar{y}, \bar{y}, \bar{x}, \bar{x}$ and (D) XY32 phase cycling - x, x, y, y, x, x, y, y, y, y, x, x, y, y, x, x, $\bar{x}, \bar{x}, \bar{y}, \bar{y}$, $\bar{x}, \bar{x}, \bar{y}, \bar{y}$, $\bar{y}, \bar{y}, \bar{x}, \bar{x}$, $\bar{y}, \bar{y}, \bar{x}, \bar{x}$, $y, y, \bar{x}, \bar{x}$, $y, y, \bar{x}, \bar{x}$, $\bar{x}, \bar{x}, y, y$, $\bar{x}, \bar{x}, y, y$, $\bar{y}, \bar{y}, x, x$, $\bar{y}, \bar{y}, x, x$, $x, x, \bar{y}, \bar{y}$, $x, x, \bar{y}, \bar{y}$.

Figure S4A shows the dependence of the simulated SPEPS curves on the number of proximate proton spins, ranging from zero (black curves) to three (green curves).

In Figure S4B, we demonstrate the numerical behavior of the SPEPS signal when assuming rf-field inhomogeneity on either one channel (nitrogen channel, I) or on both channels (nitrogen and carbon channels, II). For simulating rf-field inhomogeneity we employed the same approach as in our previous work:[5] the rf-field inhomogeneity profile was simulated with a Gaussian function. The profile of rf-field inhomogeneity in MAS probes has been investigated previously,[6–8] where the Gaussian function provided an accurate description of rf-field inhomogeneity in balanced probes. All these simulations affirm the resistance of SPEPS element against the effects of proximate protons and its small dependence on rf-field inhomogeneity.

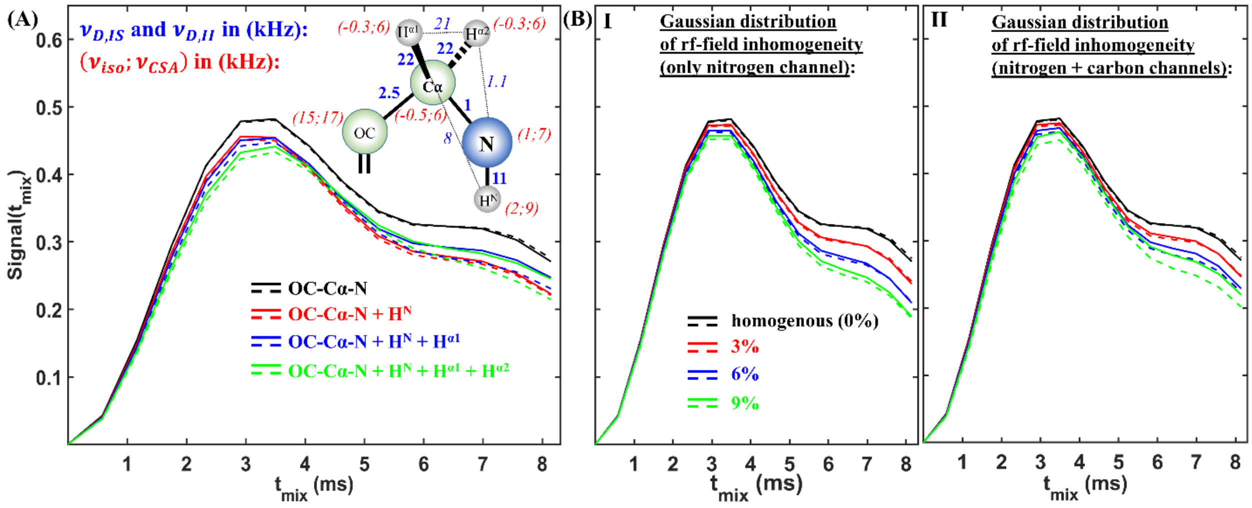

Figure S4 Numerical dependencies of SPEPS curves on number of proximate proton spins (A) and Gaussian rf-field inhomogeneity (BI-II) are shown. The solid and dashed lines represent transfer efficiencies of $C\alpha_x \rightarrow N_x$ and $C\alpha_y \rightarrow N_y$, respectively. (A) The main OC-Cα-N spin system was simulated (black curves) with the addition of one proton spin (H^N – red curves), two proton spins (H^N, $H^{\alpha 1}$ – blue curves) and three proton spins (H^N, $H^{\alpha 1}$ and $H^{\alpha 2}$ – green curves). The dipolar couplin values (ν_D), the isotropic chemical shift and and chemical shift anisotropy (ν_{iso}; ν_{CSA}) used in simulations are depicted in Figure A. (B) Gaussian rf-field inhomogeneity distribution were applied to either one channel (I – nitrogen) or both channels (II – nitrogen and carbon): 0% - black curves; 3% - red curves; 6% - blue curves and 9% - green curves. In all simulations, a 55 kHz MAS was used.

Figure S5A shows the experimental ^{15}N-^{1}H SPEPS profile. Figure S5B compares windowed SPEPS (wSPEPS, black) and SPEPS (red) transfer efficiencies as a function of mixing time. Both elements provide maximal transfer efficiency with the first mixing time point (290.09 μs). However, wSPEPS provides ~14% higher transfer efficiency than SPEPS.

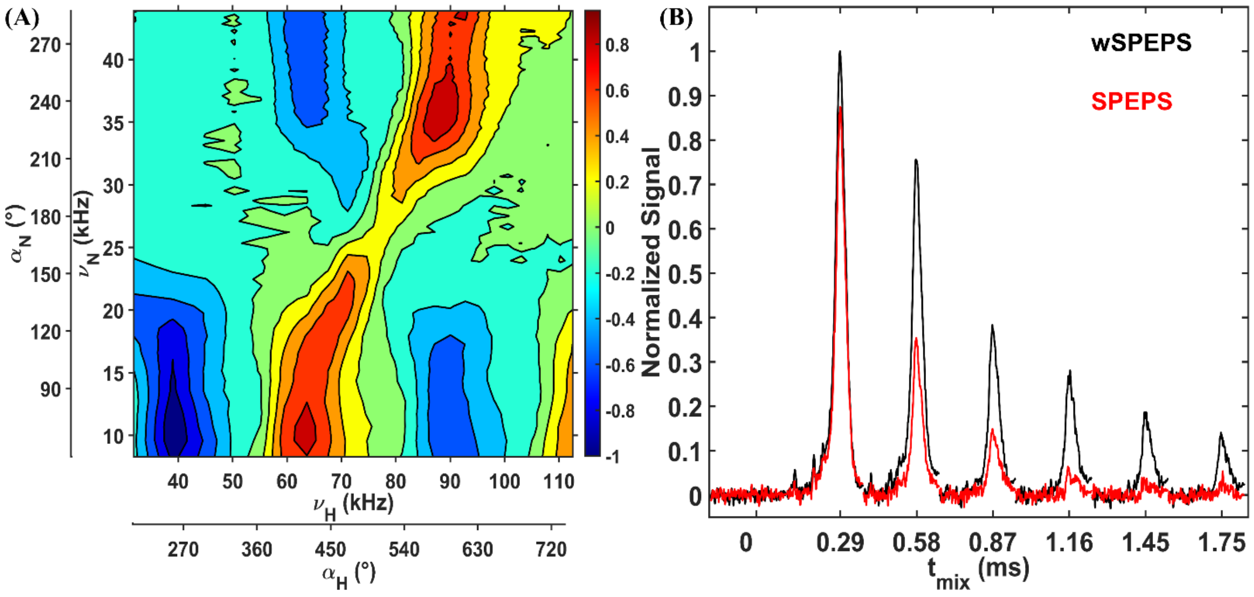

Figure S5 Experimental 1D (HN)H SPEPS $^{15}N \rightarrow {}^{1}H$ profile (A) and SPEPS, windowed SPEPS (wSPEPS) transfer efficiencies plotted against mixing times (B). (A) The SPEPS transferred signal intensity (t_{mix}=0.291 ms) is plotted as a function of applied rf-field strength (flip angle values). Both negative intensity (DQ signal) and positive intensity (ZQ) is observed. (B) SPEPS (red) and wSPEPS (black) curves. The phase cycling of SPEPS and wSPEPS pulses was (16 pulses per basis element): $y, y, x, x, y, y, x, x, \bar{x}, \bar{x}, \bar{y}, \bar{y}, \bar{x}, \bar{x}, \bar{y}, \bar{y}$. For SPEPS pulses, the length of each pulse was T_R. For wSPEPS pulses, the widths of the windows were $0.5T_R$ on both channels and the applied rf-field strengths (rf-field powers) were two times (four times) higher than for SPEPS. The length of each pulse was $0.5T_R$. Acquired at a 600 MHz spectrometer with 55 kHz MAS using a sample of ^{13}C,^{15}N labelled S31N M2.

Figure S6 compares 1D 90° ^{15}N spectrum (black) with 1D ^{13}C-^{15}N spectrum (red). ^{13}C were excited with a hard 90° pulse. The 90° ^{15}N signal was scaled with a factor of 1.47, which takes into account the ratio of the ^{13}C and ^{15}N gyromagnetic ratios (2.48) and the fraction of observed signals that can be observed in CP spectra (51/86, where 86 is the total number of amino acids in the M2 dimer). The remaining intensity ratio indicates an estimated transfer efficiency of approximately 31 percent. This estimate assumes that all signals are detected in the direct polarization spectrum. If this is incorrect, i.e. if some signals are broadened beyond detection, then the actual transfer efficiency would be lower than this estimate, potentially as low as 18 percent.

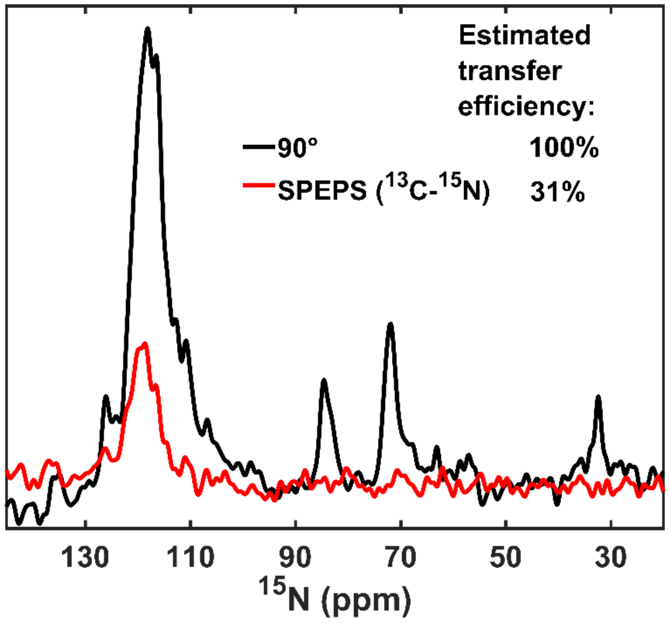

Figure S6 Comparison of 1D ^{15}N spectra acquired with a single 90° pulse (direct polarization, black) and with 1D (C)N experiment with SPEPS element for $^{13}C \rightarrow ^{15}N$ transfer. For the SPEPS experiment, ^{13}C resonances were excited with a hard 90° pulse. For the 90° and SPEPS experiments, delay times of 60 s and 12 s between scans were utilized, respectively. The spectra were acquired at a 600 MHz spectrometer with 55 kHz MAS and using the ^{13}C, ^{15}N-labelled S31N M2 sample, containing Cu^{2+} ethylenediaminetetraacetic acid.

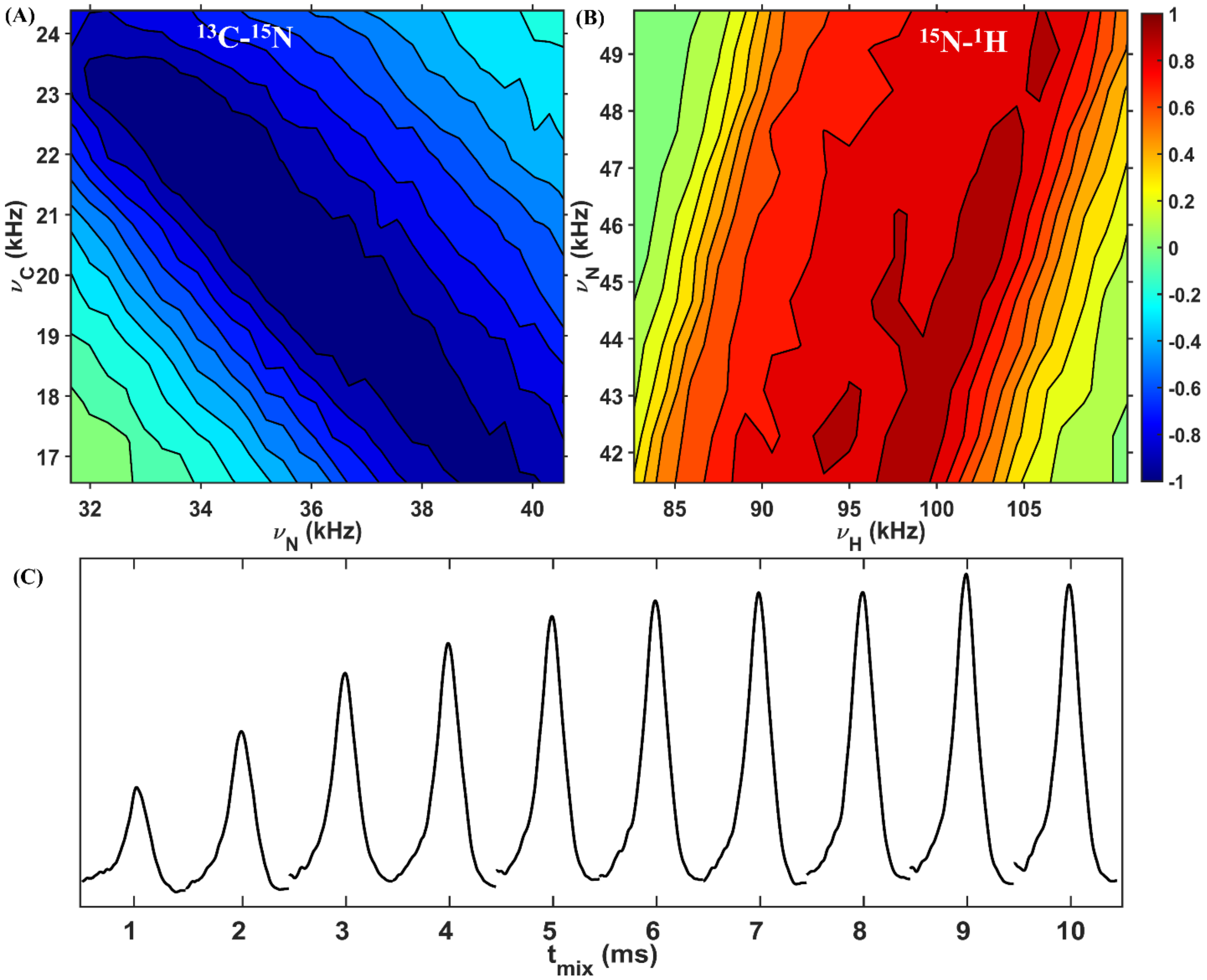

Figure S7 1D (HCAN)H CP optimization was performed, consisting of three parts: (A) $^{13}C \rightarrow ^{15}N$ transfer optimization (9 ms mixing) with [64%-94%] tangential ramp (TANG in Bruker Topspin); (B) $^{15}N \rightarrow ^{1}H$ optimization (0.8 ms mixing) with [100%-80%] linear ramp and (C) $^{13}C \rightarrow ^{15}N$ mixing time optimization. The spectra were acquired at a 600 MHz spectrometer with 55 kHz MAS using ^{13}C, ^{15}N-labelled S31N M2, containing Cu^{2+} ethylenediaminetetraacetic acid to accelerate the acquisition.

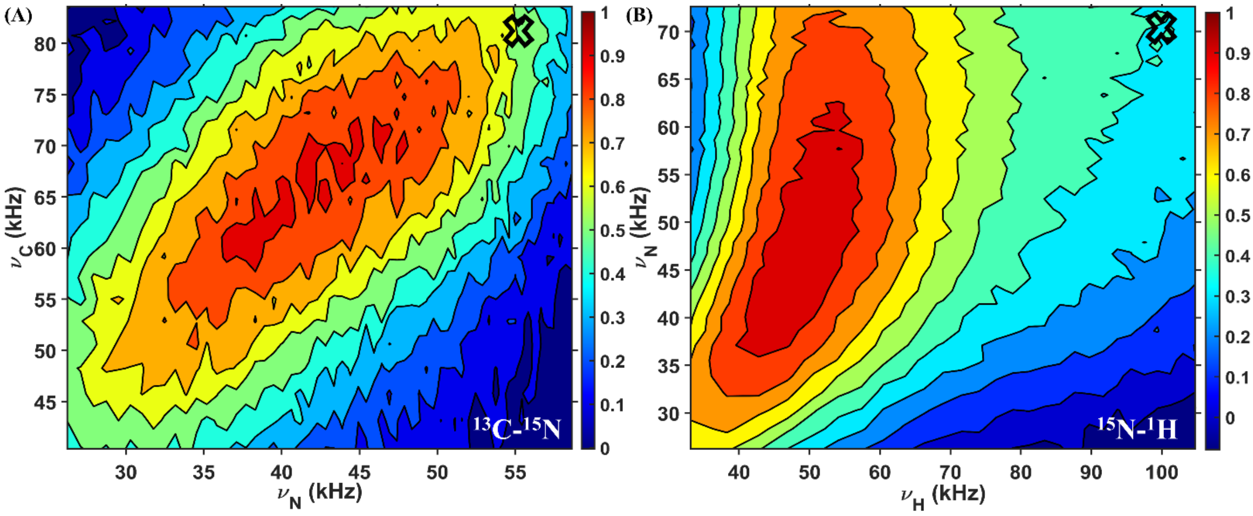

Figure S8 1D (HCAN)H TROP optimization was performed, consisting of two parts: (A) $^{13}C \rightarrow ^{15}N$ transfer optimization (3.636 ms mixing), and (B) $^{15}N \rightarrow ^{1}H$ optimization (0.8 ms mixing). Black crosses indicate the recommended rf-field strength values.[9] Profiles were acquired at a 600 MHz spectrometer with 55 kHz MAS using a sample of S31N M2.

Figure S9 compares 1D spectra, obtained with SPEPS (black), TROP (magenta) and CP (cyan) elements for $^{13}C \rightarrow ^{15}N$ transfer under different experimental conditions and samples: S31N M2 at 600 MHz and 55 kHz MAS (A) and wt M2 at 950 MHz and 100 kHz MAS (B). Both samples are non-deuterated. For (A) SPEPS exhibits higher 1D signal after ^{13}C-^{15}N transfer compared to TROP and CP (SPEPS: TROP: CP – 1: 0.81: 0.85, A). Since only one of two possible components are detected in the 1D spectra, the expected transfer efficiency for a 3D would be (SPEPS: TROP: CP – 1.4: 1.15: 0.85). Under ultra-fast MAS rate of 100 kHz (B), SPEPS and CP provide similar transfer efficiency. The TROP element was not investigated under 100 kHz MAS due to the limitations of the rf-field powers of the probe, and since a suitable TROP pulse has not been reported for 100 kHz MAS.

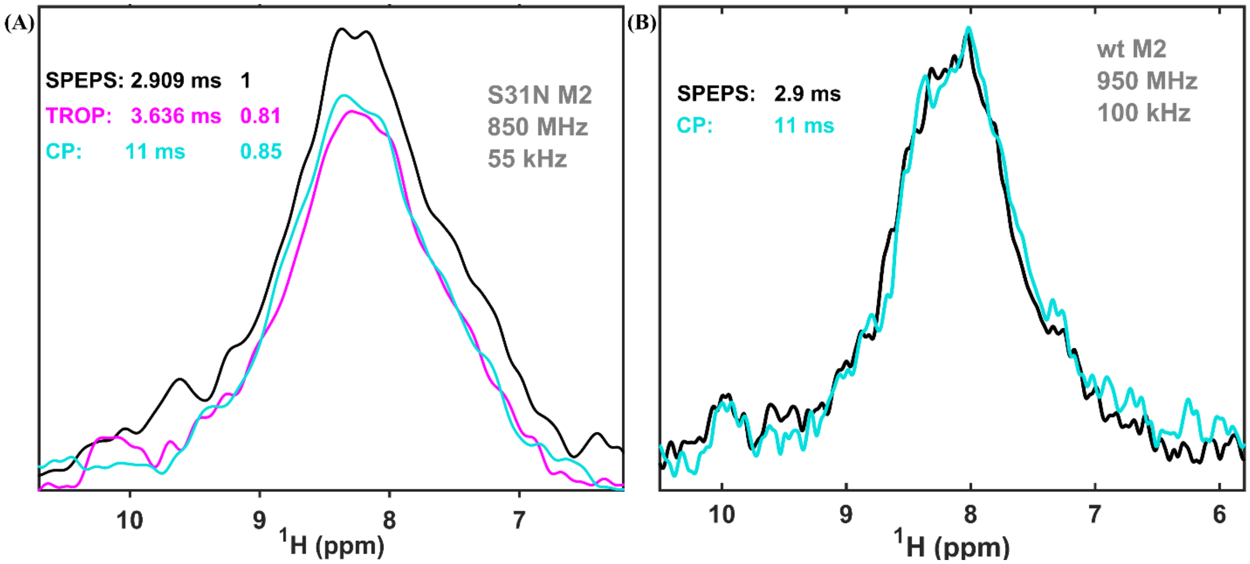

Figure S9 1D (HCAN)H experiment comparing different elements for $^{13}C \rightarrow ^{15}N$ transfer. Comparisons of SPEPS (black), TROP (magenta) and CP (cyan) spectra at 850 MHz, 55 kHz MAS and using a sample of ^{13}C, ^{15}N-labelled S31N M2, containing Cu^{2+} ethylenediaminetetraacetic acid (A) and at 950 MHz, 100 kHz and using a sample of ^{13}C, ^{15}N-labelled M2 (B). Assuming no indirect phase correction is needed, one of two transverse components is detected since there was no chemical shift evolution of indirect dimensions. Therefore, both SPEPS and TROP are expected to benefit by an additional factor of 1.4 beyond what is shown in the figure. The polarization transfer conditions for $^{1}H \rightarrow ^{13}C$ and $^{15}N \rightarrow ^{1}H$ transfers were the same for all three methods (ramped CP).

Figure S10 compares 1D spectra, obtained with SPEPS (black), TROP (magenta) and CP (cyan) elements for $^{15}N \rightarrow ^{1}H$ transfers. For ^{15}N-^{1}H transfer, CP outperforms both SPEPS and TROP elements in terms of transfer efficiency (1D intensity of SPEPS: TROP: CP – 1: 0.7: 1.89, expected 3D efficiency 1.4:1:1.89). Therefore, we used standard CP for multidimensional spectra and focused our further investigations on ^{13}C-^{15}N CP, SPEPS and TROP transfers.

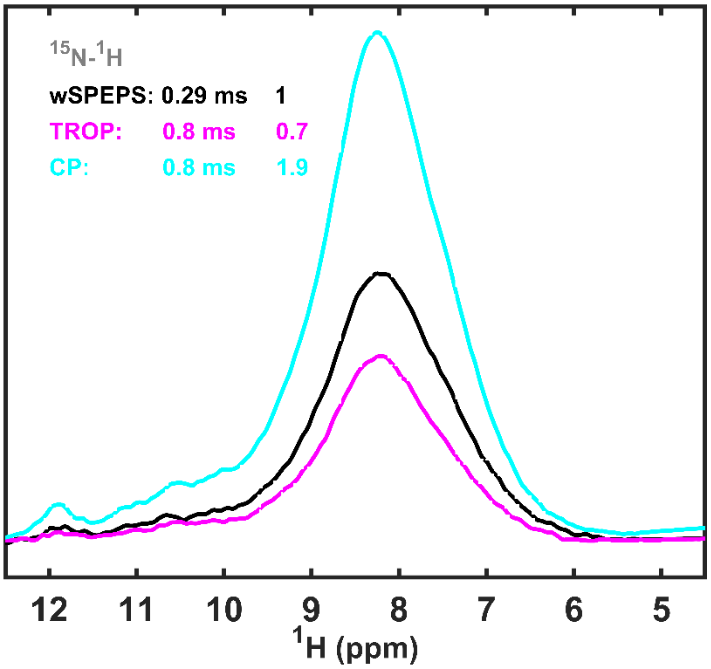

Figure S10 Comparisons of 1D (HN)H spectra for SPEPS (black), TROP (magenta) and CP (cyan) $^{15}N \rightarrow ^{1}H$ transfers. Assuming no indirect phase correction is needed, one of two transverse components is detected since there was no chemical shift evolution of indirect dimensions. Both SPEPS and TROP are expected to benefit by an additional factor of 1.4 beyond what is shown in the figure. The polarization transfer conditions for the initial $^{1}H \rightarrow ^{15}N$ transfer were the same for all three methods ([80%-100%] ramped CP with 800 μs mixing time). The spectra were acquired at a 600 MHz spectrometer with 55 kHz MAS, using a sample of ^{13}C, ^{15}N-labelled S31N M2, containing Cu^{2+} ethylenediaminetetraacetic acid.

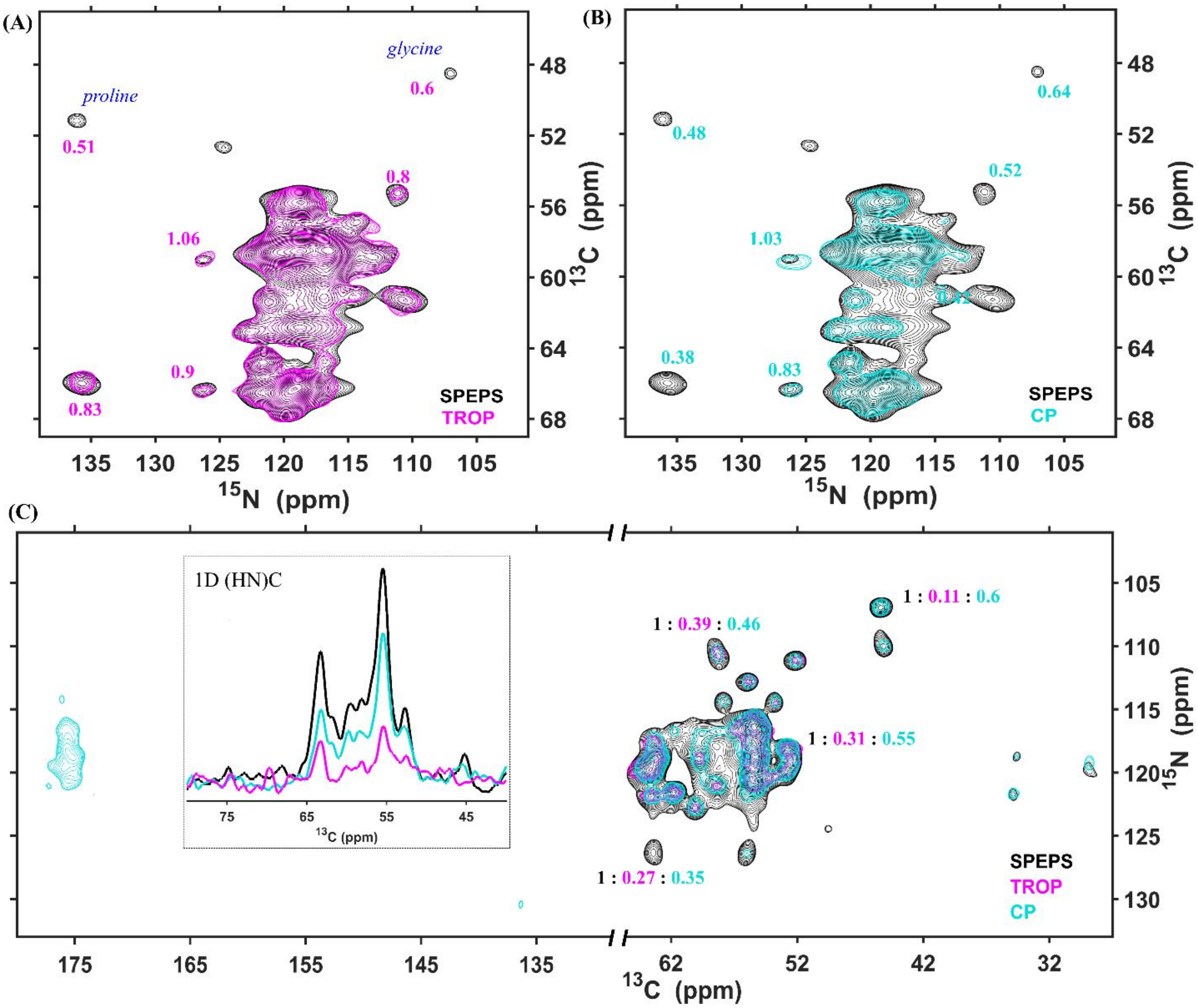

Figure S11 2D (H)CAN (A-B) and (H)NC (C) spectra with SPEPS (black), TROP (magenta) and CP (cyan) recoupling elements for $^{13}C \rightarrow ^{15}N$ (A-B) and $^{15}N \rightarrow ^{13}C$ (C) transfers. The polarization transfer for $^{1}H \rightarrow ^{15}N$ (A-B) and for $^{1}H \rightarrow ^{13}C$ (C) utilized CP. The inset in (C) compares 1D (HN)C spectra with SPEPS, CP and TROP elements. Numbers indicate relative intensities scaled to the intensities of the SPEPS spectrum. The spectra were acquired at a, 850 MHz (A-B) or a 600 MHz (C) spectrometers with 55 kHz MAS and using the S31N M2 sample, containing Cu^{2+} ethylenediaminetetraacetic acid to accelerate the acquisition. The carbon and nitrogen carrier frequencies were set to 53.7 ppm and 116 ppm (A-B) and 48 ppm and 113 ppm (C), respectively. Further experimental details in the experimental methods, below.

EXPERIMENTAL METHODS

Solid state NMR spectroscopy

Figures S13-S15 show 2D (H)NC, 2D (H)CAN, 3D (H)CANH and 3D (H)CANCO pulse sequences. There are no special requirements for the positions of the carrier frequencies (^{13}C and ^{15}N CF). However, large offset values affect the SPEPS transfer efficiency.

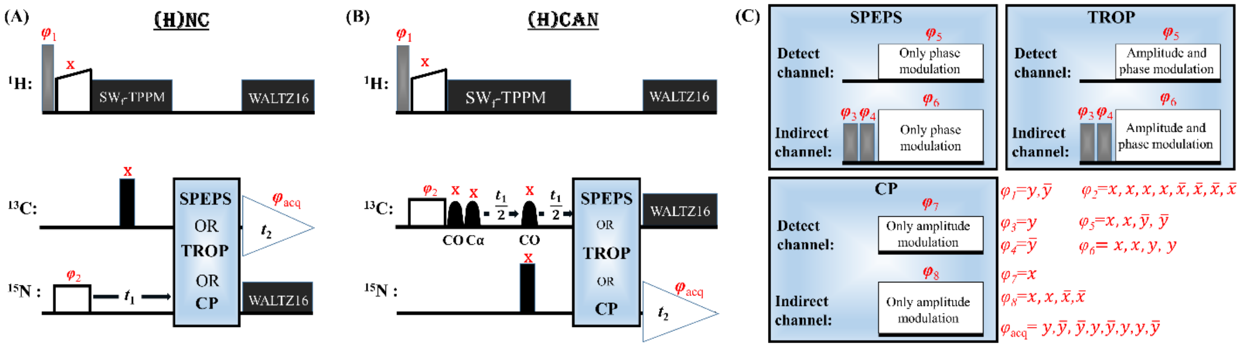

Figure S12 2D (H)NC (A) and (H)CAN (B) with either SPEPS, TROP or CP (C) recoupling eleements. π/2-pulses are indicated by light rectangles and π-pulses are by black rectangles. All phase cycling information is shown in the Figure. The ramped CP transfers from proton to carbon are depicted with a constant power on the carbon channel and a power ramp on the proton channel. During the indirect dimension (t_1) and acquisition (t_2), SW_f-TPPM decoupling[14] is applied. π-pulses in the middle of t_1 and t_2 are used to decouble carbon-nitrogen interactions. REBURP soft selective pulses[15] are applied on carbon channel to select ^{13}CA spins. For $^{13}C \rightarrow ^{15}N$ transfers, TROP shaped pulse lists were taken from Ref.[9]; tangential (TANG) shape was used for CP;[16] SPEPS was used as the shaped pulse list (shown in 'Bruker Pulse Program' section) with constant amplitude. For SPEPS and TROP elements, a pair of π/2-pulses is applied before the transfer in order to invert the real part of the signal for even increments of the indirect dimension.

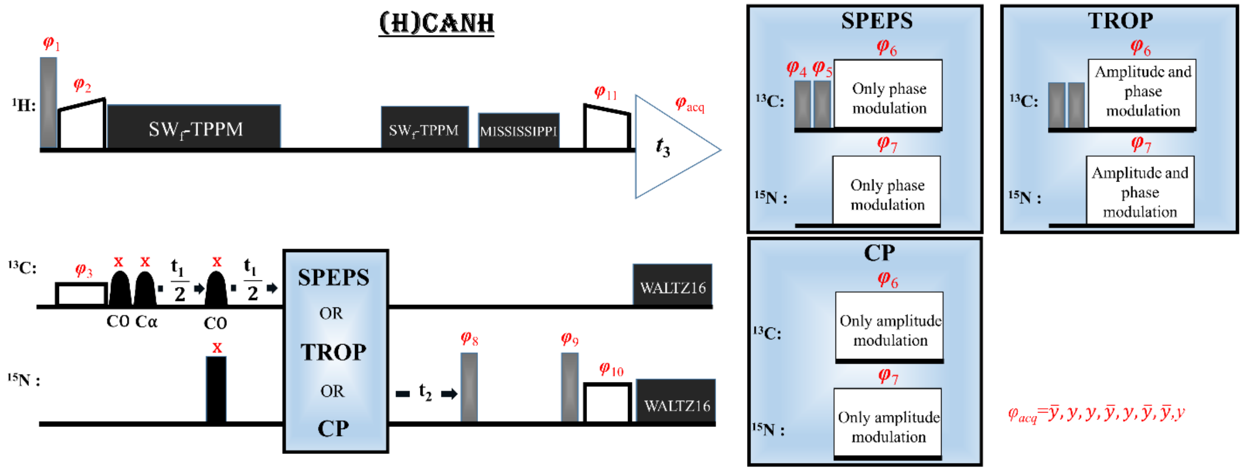

Figure S13 3D (H)CANH with either SPEPS, TROP or CP (C) recoupling eleements for ^{13}CA→^{15}N transfer. π/2-pulses are indicated by light rectangles and π-pulses by black rectangles. For CP, the phases are: $\varphi_1 = x$; $\varphi_2 = y, y, \bar{y}, \bar{y}$; $\varphi_3 = x$; $\varphi_6 = x, \bar{x}$; $\varphi_7 = \bar{y}$; $\varphi_8 = \varphi_9 = x$; $\varphi_{10} = 4 \times (y), 4 \times (\bar{y})$; $\varphi_{11} = y$. For SPEPS and TROP elements, the phases are: $\varphi_1 = y$; $\varphi_2 = x, x, \bar{x}, \bar{x}$; $\varphi_3 = \bar{x}$; $\varphi_4 = y$; $\varphi_5 = \bar{y}$; $\varphi_6 = x, \bar{y}$; $\varphi_7 = x, y$; $\varphi_8 = y$; $\varphi_9 = \bar{y}$; $\varphi_{10} = 4 \times (\bar{x}), 4 \times (x)$; $\varphi_{11} = x$. Ramped CP is used for proton to carbon transfer. During the indirect dimension (t_1) and acquisition (t_2), SW_f-TPPM decoupling[14] is applied. π-pulses in the middle of t_1 and t_2 are used to decouble carbon-nitrogen interactions. REBURP soft selective pulses[15] are applied on carbon channel to select ^{13}CA spins. During acquisition, WALTZ16 decoupling[17] was applied on nitrogen and carbon channels. For ^{13}C→^{15}N transfers, TROP shaped pulse lists were taken from Ref.[9]; tangential (TANG) shape was used for CP;[16] SPEPS was implemented as a shaped pulse list with constant amplitude modulation (shown in 'Bruker Pulse Program' section). For SPEPS and TROP elements, a pair of π/2-pulses is applied before the transfer in order to invert the real part of the signal for even increments of the indirect dimension.

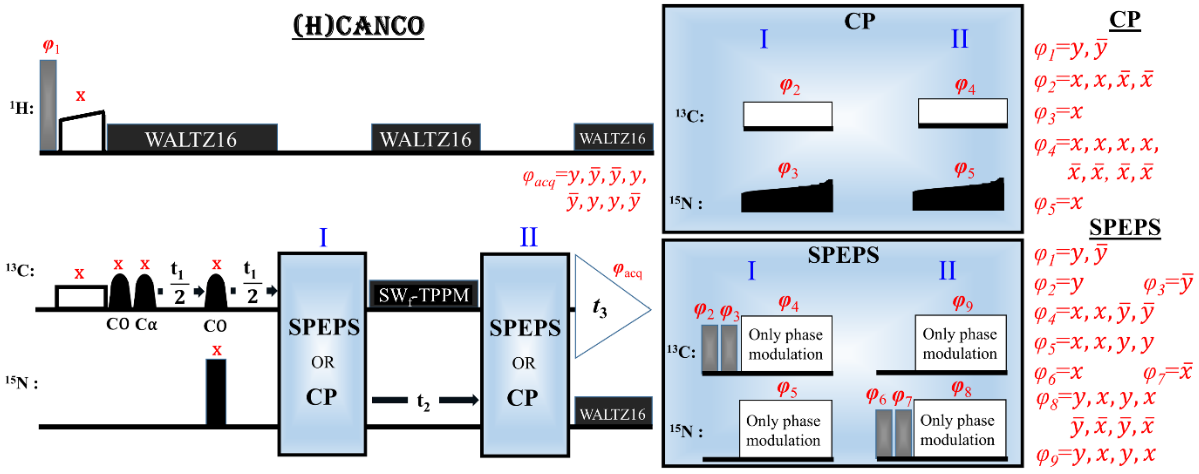

Figure S14 3D (H)CANCO pulse sequence with either SPEPS or CP recoupling eleements for ^{13}CA→^{15}N and ^{15}N→^{13}C transfers. π/2-pulses are indicated by light, π-pulses by black rectangles. Ramped CP is used for proton to carbon transfer. During the indirect dimension (t_1) and acquisition (t_2), WALTZ16 decoupling[17] and SW$_f$-TPPM decoupling[14] are applied. A single π-pulse in the middle of t_1 is used to decouble carbon-nitrogen interactions. REBURP soft selective pulses[15] are applied on carbon channel to select ^{13}CA spins. During acquisition, WALTZ16 decoupling[17] was applied on nitrogen and proton channels. For ^{13}C→^{15}N and ^{15}N→^{13}C transfers, a tangential (TANG) shape was used for CP; SPEPS was implemented as a shaped pulse list with constant amplitude. For SPEPS elements, two pairs of π/2-pulses are applied to selectively invert the real part (before ^{13}CA→^{15}N transfer) or the imaginary part (before ^{13}N→^{13}C transfer) of the signal.

600 MHz: 1D (HCAN)H, 2D (H)NC and 3D (H)CANCO experiments were acquired on a Bruker Avance III HD spectrometer operating at 14.1 T (600 MHz ^{1}H frequency) using a DVT600W2 BL1.3 mm HXY probe. The experiments were performed at 55 kHz MAS, and the temperature of the nitrogen cooling gas set to 245 K with 1000 to 1300 liters per hour. For decoupling of the heteronuclear dipolar interactions SW$_f$-TPPM[14] or WALTZ-16[17] were used on the proton channel, and WALTZ-16[17] was used on heteronuclear channels. MISSISSIPPI[18] water suppression was applied for proton detected experiments.

For ^{13}C↔^{15}N CP transfers the tangential shape was applied.[16] Table S1 summarizes the input topspin parameter for the shape:

Size [pt]	1000	Modulation Amplitude	60000.0
Offset [pt]	0	Phase [°]	70.0
Pulse length [µs]	1000	Mean amplitude	30000.0
Rotational angle [°]	90.0	Phase amplitude factor	76.92
		Dipolar coupling	2183.82

Table S1 The summary of the input parameters for generation of the TANG ramp, according to Ref.[16] and implemented in Topspin 3.5.

S31N M2 sample

Table S2 summarizes the experimental parameters of recoupled elements in 2D S31N M2 (H)NC experiments. Additional parameters are shown in Figure S15.

**2D (H)NC**		CP	TROP	SPEPS
$^{1}H \rightarrow ^{15}N$	$\nu_{rf}(^{1}H)$	linear ramp: [80%:100%] [90:112] (kHz)	linear ramp: [80%:100%] [90:112] (kHz)	linear ramp: [80%:100%] [90:112] (kHz)
	$\nu_{rf}(^{15}N)$	42 kHz	42 kHz	42 kHz
	t_{mix}	0.45 ms	0.45 ms	0.45 ms
**$^{15}N \rightarrow ^{13}C$**	**$\nu_{rf}(^{15}N)$**	**TANG ramp:[63%:94%] [28:41.8] (kHz)**	**Maximal rf-field Strength: 41.5 kHz**	**41.5 kHz**
	**$\nu_{rf}(^{13}C)$**	**20.8 kHz**	**Maximal rf-field Strength: 64.1 kHz**	**13.1 kHz**
	**t_{mix}**	**9 ms**	**3.63636 ms**	**2.90909 ms**

Table S2 The shapes, nominal rf-field strengths and transfer times for recoupling elements as implemented for ^{1}H-^{15}N and ^{15}N-^{13}C transfers at 55 kHz MAS. For ramped CP, rectangular brackets indicate the minimal and maximal rf-field of ramped rf-field strengths. For TROP, the TROP shape files were downloaded from https://optimal-nmr.net/sequences.html and used without any modification. In the table the maximal TROP rf-field strengths are shown. For all elements, rf-field strength values and mixing times indicate the experimentally determined optimum.

In Figure S15, below: SW – spectral width; TD – the number of points in the FID; IN_F – an increment time; AQ – the acquisition time. 4 Dummy Scans were used.

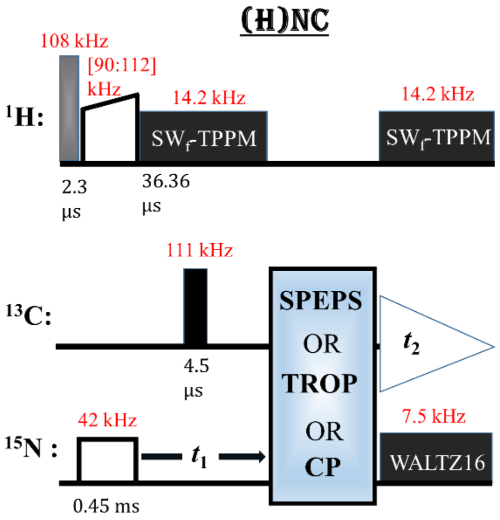

(H)NC	F2	F1
	^{13}C	^{15}N
TD	1600	360
SW(ppm)	395.1171	180
IN_F(μs)		91.51
AQ(s)	0.01344	0.0164712
CF	48	113

Number Scans: 96
Recycle delay: 0.6 s
Total Time of the experiment: 6.03 hours

Figure S15 The 2D (H)NC sequence and the experimental parameters (**Figure S11C in the SI**). Red numbers represent rf-field power in kHz. The hard pulse durations are shown in μs, while total duration of CP and decoupling are in ms. For ^{1}H→^{15}N transfers CP with [80%:100%] ramp was applied on the proton channel. The details of ^{1}H→^{15}N and ^{15}N→^{13}C transfers (the shapes, the rf-field strengths and mixing times) are summarized in Table S2.

Table S3 summarizes the experimental parameters of recoupling elements used for the 3D S31N M2 (H)CANCO spectrum. Additional parameters are shown in Figure S16.

**3D (H)CANCO**		CP	SPEPS
^{1}H→^{13}CA	$\nu_{rf}(^{1}H)$	linear ramp: [80%:100%] [59:74] (kHz)	linear ramp: [80%:100%] [59:74] (kHz)
	$\nu_{rf}(^{13}C)$	16 kHz	16 kHz
	t_{mix}	0.45 ms	0.45 ms
**^{13}CA→^{15}N**	**$\nu_{rf}(^{15}N)$**	**TANG ramp:[63%:94%] [28:41.8] (kHz)**	**41.5 kHz**
	**$\nu_{rf}(^{13}C)$**	**20.8 kHz**	**13.1 kHz**

	**t_{mix}**	**9 ms**	**2.90909 ms**
^{15}N→^{13}CO	ν_{rf}(^{15}N)	TANG ramp:[63%:94%] [28:41.8] (kHz)	41.5 kHz
	ν_{rf}(^{13}C)	20.8 kHz	13.1 kHz
	t_{mix}	9 ms	2.90909 ms

Table S3 The shapes, nominal rf-field strengths and transfer times for recoupling elements as implemented for ^{1}H-^{13}CA, ^{13}CA-^{15}N and ^{15}N-^{13}CO transfers at 55 kHz MAS. For ramped CP, rectangle brackets indicate the minimal and maximal rf-field of ramped rf-field strengths. For all elements, rf-field strengths values and mixing times indicate the experimentally determined optimum.

In Figure S16, below: SW – spectral width; TD – the number of points in the FID; IN_F – an increment time; AQ – the acquisition time. 4 Dummy Scans were used.

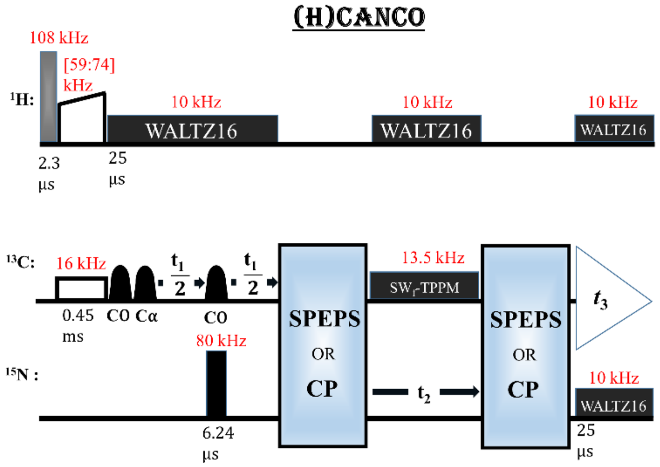

(H)CANCO	F3	F2	F1
	^{13}CO	^{15}N	^{13}CA
TD	1898	116	100
SW(ppm)	395.0626	85	70
IN_F(µs)		193.78	94.82
AQ(s)	0.0159432	0.0112391	0.0047408
CF	186	115	60.71

Number Scans: 32
Recycle delay: 0.5 s
Total Time of the experiment: 2.36 days

Figure S16 The 3D (H)CANCO sequence and the experimental parameters (**Figure 5 in the main text**). Red numbers represent rf-field power in kHz. The hard pulse durations are shown in µs, while total duration of CP and decoupling are in ms. For ^{1}H→^{13}C transfers, SPECIFIC-CP conditions[19,20] with [80%:100%] ramp was applied on the proton channel. For ramped CP, rectanglular brackets indicate the minimal and maximal rf-field of ramped rf-field strengths. The details of ^{1}H→^{13}CA, ^{13}CA→^{15}N and ^{15}N→^{13}CO transfers (the shapes, the rf-field strengths and mixing times) are summarized in Table S3.

950 MHz: 3D (H)CANH experiments were acquired on a Bruker Avance III HD spectrometer operating at 22.3 T (950 MHz ^{1}H frequency), equipped with a 0.7 mm HCDN MAS probe at 100 kHz MAS. The temperature of the nitrogen cooling gas set to 252 K with 550 liters per hour. For decoupling of the heteronuclear dipolar interactions SW$_f$-TPPM[14] was used on the proton channel, and WALTZ-16[17] was used on heteronuclear channels. MISSISSIPPI[18] water suppression was applied for proton detected experiments.

wt M2 sample

Table S4 summarizes the experimental parameters of recoupling elements used for the 3D (H)CANH spectrum of wt M2. Additional parameters are shown in Figure S17.

**3D (H)CANH**		CP	SPEPS
^{1}H→^{13}CA	ν_{rf}(^{1}H)	linear ramp: [80%:100%]<br>[108:135] (kHz)	linear ramp: [80%:100%]<br>[108:135] (kHz)
	ν_{rf}(^{13}C)	25 kHz	25 kHz
	t_{mix}	0.5 ms	0.5 ms
**^{13}CA→^{15}N**	**ν_{rf}(^{15}N)**	**26 kHz**	**24 kHz**
	**ν_{rf}(^{13}C)**	**TANG ramp:[63%:94%]<br>[59:88] (kHz)**	**75 kHz**
	**t_{mix}**	**10 ms**	**2.56 ms**
^{15}N→^{1}H	ν_{rf}(^{1}H)	linear ramp: [80%:100%]<br>[106:132] (kHz)	linear ramp: [80%:100%]<br>[106:132] (kHz)
	ν_{rf}(^{15}N)	25 kHz	25 kHz
	t_{mix}	0.8 ms	0.8 ms

Table S3 The shapes, nominal rf-field strengths and transfer times for recoupling elements as implemented for ^{1}H-^{13}CA, ^{13}CA-^{15}N and ^{15}N-^{1}H transfers at 100 kHz MAS. For ramped CP, rectangle brackets indicate the minimal and

maximal rf-field of ramped rf-field strengths. For all elements, rf-field strengths values and mixing times indicate the experimentally determined optimum.

In Figure S17, below: SW – spectral width; TD – the number of points in the FID; IN_F – an increment time; AQ – the acquisition time. 4 Dummy Scans were used.

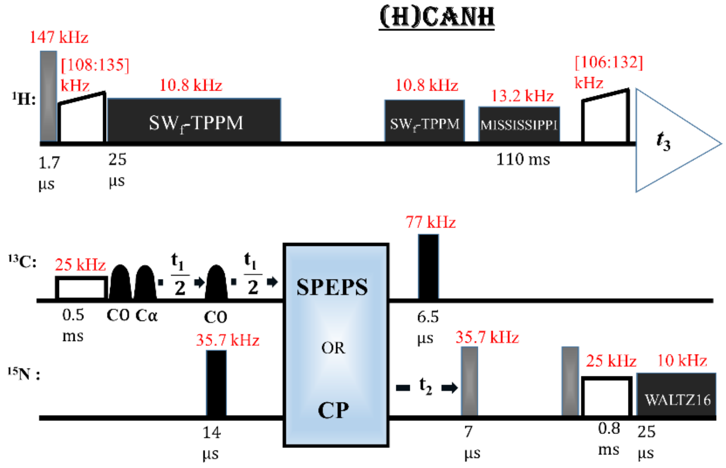

(H)CANH	F3	F2	F1
	^{1}H	^{15}N	^{13}C
TD	4096	80	80
SW(ppm)	52.6103	60	60
IN_F(µs)		173.05	69.74
AQ(s)	0.04096	0.0069219	0.0027896
CF	4.7	116	50.5

Number Scans: 8
Recycle delay: 1.6 s
Total Time of the experiment: 1.05 days

Figure S17 The 3D (H)CANH sequence and the experimental parameters (**Figures 2B, E in the main text**). Red numbers represent rf-field power in kHz. The hard pulse durations are shown in µs, while total duration of CP and decoupling are in ms. For ^{1}H→^{13}C transfers, CP with [80%:100%] ramp was applied on the proton channel. For ^{15}N→^{1}H transfers, CP with [80%:100%] ramp was applied on the proton channel. For ramped CP, rectangular brackets indicate the minimal and maximal rf-field of ramped rf-field strengths. The details of ^{1}H→^{13}CA, ^{13}CA→^{15}N and ^{15}N→^{1}H transfers (the shapes, the rf-field strengths and mixing times) are summarized in Table S4.

850 MHz: 2D (H)CAN and 3D (H)CANH experiments were acquired on a Avance III spectrometer operating at 19.97 T (850 MHz ^{1}H field strength), equipped with a 1.3 mm HCN MAS probe at 55 kHz MAS. The temperature of the nitrogen cooling gas was set to 245 K. Heteronuclear dipolar interactions were decoupled with SW_f-TPPM,[14] (proton channel) and WALTZ-16,[17] (nitrogen channel). For water suppression, MISSISSIPPI[18] was used.

S31N M2 sample

Table S5 summarize the experimental parameters of recoupling elements in 2D S31N M2 (H)CAN experiments. Additional parameters are shown in Figure S18.

**2D (H)CAN**		CP	TROP	SPEPS
^{1}H→^{13}CA	ν_{rf}(^{1}H)	linear ramp: [80%:100%] [88:110] (kHz)	linear ramp: [80%:100%] [88:110] (kHz)	linear ramp: [80%:100%] [88:110] (kHz)
	ν_{rf}(^{13}C)	48 kHz	48 kHz	48 kHz
	t_{mix}	0.68 ms	0.68 ms	0.76 ms
**^{13}CA→^{15}N**	**ν_{rf}(^{15}N)**	**TANG ramp:[63%:94%] [27.8:41.4] (kHz)**	**Maximal rf-field Strength: 40 kHz**	**41.4 kHz**
	**ν_{rf}(^{13}C)**	**17.8 kHz**	**Maximal rf-field Strength: 61 kHz**	**10.5 kHz**
	**t_{mix}**	**11 ms**	**3.63636 ms**	**2.90909 ms**

Table S5 The shapes, nominal rf-field strengths and transfer times for recoupling elements as implemented for ^{1}H-^{13}CA and ^{13}CA-^{15}N transfers at 55 kHz MAS. For ramped CP, rectanglular brackets indicate the minimal and maximal rf-field of ramped rf-field strengths. For TROP, the TROP shape files were downloaded from https://optimal-nmr.net/sequences.html and used without any modification. In the table the maximal TROP rf-field strengths are shown. For all elements, rf-field strengths values and mixing times indicate the experimentally determined optimum.

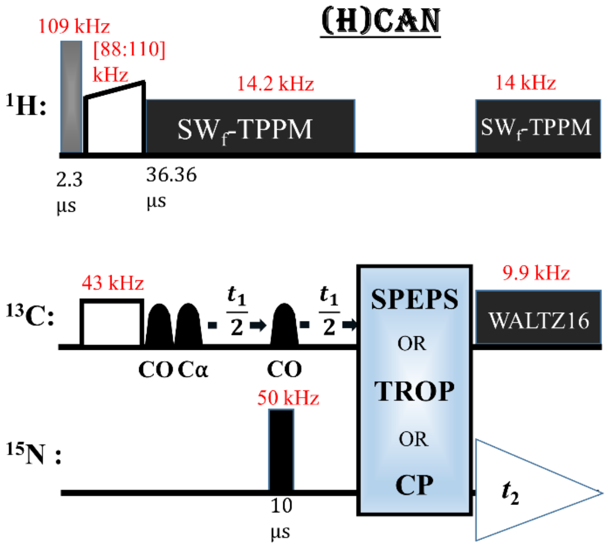

(H)CAN	F2	F1
	^{15}N	^{13}C
**TD**	1800	700
**SW(ppm)**	682.6841	300
**IN_F(μs)**		15.5904
**AQ(s)**	0.0153	0.0054566
**CF**	116	53.7

Number Scans: 80
Recycle delay: 0.4 s
Total Time of the experiment: 6.64 hours

Figure S18 The 2D (H)CAN sequence and the experimental parameters (**Figures S11A-B in the SI**). Red numbers represent rf-field power in kHz. The hard pulse durations are shown in μs, while total duration of CP and decoupling are in ms. For ^{1}H→^{13}CA transfers, CP with [80%:100%] ramp was applied on the proton channel. The details of ^{1}H→^{13}CA, ^{1}H→^{15}N and ^{13}C→^{15}N transfers (the shapes, the rf-field strengths and mixing times) are summarized in Table S5.

Table S6 summarizes the experimental parameters of recoupling elements used for the 3D (H)CANH spectrum of S31N M2. Additional parameters are shown in Figure S19.

**3D (H)CANH**		CP	TROP	SPEPS
^{1}H→^{13}CA	$\nu_{rf}(^{1}H)$	linear ramp: [80%:100%] [88:110] (kHz)	linear ramp: [80%:100%] [88:110] (kHz)	linear ramp: [80%:100%] [88:110] (kHz)
	$\nu_{rf}(^{13}C)$	43 kHz	43 kHz	43 kHz
	t_{mix}	0.52 ms	0.68 ms	0.76 ms
**^{13}CA→^{15}N**	$\nu_{rf}(^{15}N)$	**TANG ramp:[63%:94%] [27.8:41.4] (kHz)**	**Maximal rf-field Strength: 40 kHz**	**41.4 kHz**

	ν_{rf}(^{13}C)	**17.8 kHz**	**Maximal rf-field Strength: 61 kHz**	**10.5 kHz**
	t_{mix}	**11 ms**	**3.63636 ms**	**2.90909 ms**
^{15}N→^{1}H	ν_{rf}(^{1}H)	linear ramp: [100%:80%] [104:83] (kHz)	linear ramp: [100%:80%] [104:83] (kHz)	linear ramp: [100%:80%] [104:83] (kHz)
	ν_{rf}(^{15}N)	43 kHz	43 kHz	43 kHz
	t_{mix}	0.8 ms	1 ms	0.88 ms

Table S5 The shapes, nominal rf-field strengths and transfer times for recoupling elements as implemented for ^{1}H-^{13}CA, ^{13}CA-^{15}N and ^{15}N-^{1}H transfers at 55 kHz MAS. For ramped CP, rectanglular brackets indicate the minimal and maximal rf-field of ramped rf-field strengths. For TROP, the TROP shape files were downloaded from https://optimal-nmr.net/sequences.html and used without any modification. In the table the maximal TROP rf-field strengths are shown. For all elements, rf-field strengths values and mixing times indicate the experimentally determined optimum.

In Figure S19, below: SW – spectral width; TD – the number of points in the FID; IN_F – an increment time; AQ – the acquisition time. 2 Dummy Scans were used.

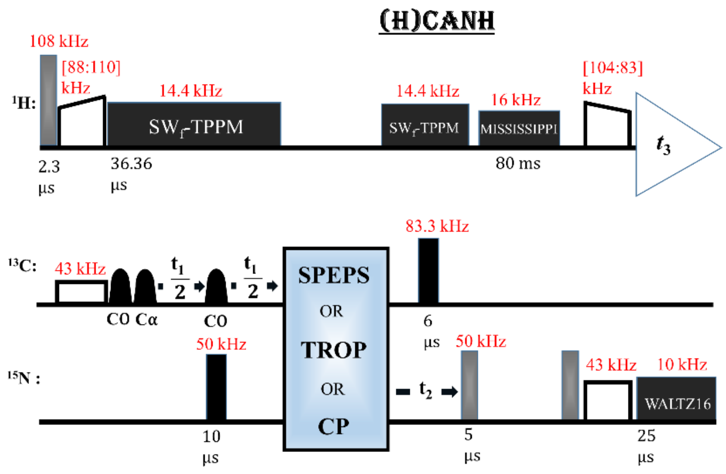

(H)CANH	F3	F2	F1
	^{1}H	^{15}N	^{13}C
TD	1800	110	126
SW(ppm)	50.6948	50	48
IN_F(µs)		232.1126	97.4399
AQ(s)	0.02088	0.0127662	0.0061387
CF	4.7	116	53.7

Number Scans: 8
Recycle delay: 0.4 s
Total Time of the experiment: 16.02 hours

Figure S19 The 3D (H)CANH sequence and the experimental parameters (Figures 2 in the main text). Red numbers represent rf-field power in kHz. The hard pulse durationss are shown in µs, while total duration of CP and

decoupling are in ms. For $^{1}H \rightarrow ^{13}C$ transfers, CP with [80%:100%] ramp was applied on the proton channel. For $^{15}N \rightarrow ^{1}H$ transfers, CP with [100%:80%] ramp was applied on the proton channel. For ramped CP, rectanglular brackets indicate the minimal and maximal rf-field of ramped rf-field strengths. The details of $^{1}H \rightarrow ^{13}CA$, $^{13}CA \rightarrow ^{15}N$ and $^{15}N \rightarrow ^{1}H$ transfers (the shapes, the rf-field strengths and mixing times) are summarized in Tables S6.

A-Synuclein fibril sample

Table S7 summarizes the experimental parameters of recoupling elements used for the 3D (H)CANH spectrum of αSYN. Additional parameters are shown in Figure S20.

**3D (H)CANH**		CP	SPEPS
$^{1}H \rightarrow ^{13}CA$	$\nu_{rf}(^{1}H)$	linear ramp: [80%:100%] [61:76] (kHz)	linear ramp: [80%:100%] [61:76] (kHz)
	$\nu_{rf}(^{13}C)$	11.5 kHz	11.5 kHz
	t_{mix}	0.66 ms	0.48 ms
**$^{13}CA \rightarrow ^{15}N$**	$\boldsymbol{\nu_{rf}(^{15}N)}$	**TANG ramp:[63%:94%] [26.3:41.8] (kHz)**	**41 kHz**
	$\boldsymbol{\nu_{rf}(^{13}C)}$	**13.4 kHz**	**11.5 kHz**
	$\boldsymbol{t_{mix}}$	**14 ms**	**2.90909 ms**
$^{15}N \rightarrow ^{1}H$	$\nu_{rf}(^{1}H)$	linear ramp: [100%:80%] [103:83] (kHz)	linear ramp: [100%:80%] [103:83] (kHz)
	$\nu_{rf}(^{15}N)$	40 kHz	40 kHz
	t_{mix}	0.7 ms	0.8 ms

Table S6 The nominal rf-field strengths and transfer times for recoupling elements as implemented for ^{1}H-^{13}CA, ^{13}CA-^{15}N and ^{15}N-^{1}H transfers at 55 kHz MAS. For ramped CP, rectanglular brackets indicate the minimal and maximal rf-field of ramped rf-field strengths. For all elements, rf-field strengths values and mixing times indicate the experimentally determined optimum.

In Figure S20, below: SW – spectral width; TD – the number of points in the FID; IN_F – an increment time; AQ – the acquisition time. 8 Dummy Scans were used.

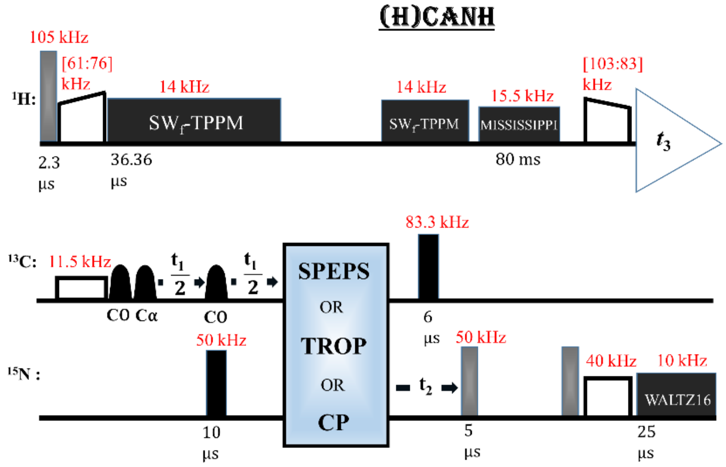

(H)CANH	F3	F2	F1
	^{1}H	^{15}N	^{13}C
TD	1800	82	74
SW(ppm)	50.6948	40	30
IN_F(µs)		290.1407	155.9038
AQ(s)	0.02088	0.0118958	0.0057684
CF	4.7	116	53.7

Number Scans: 8
Recycle delay: 1.2 s
Total Time of the experiment: 17.78 hours

Figure S20 The 3D (H)CANH sequence and the experimental parameters (**Figure 4 in the main text**). Red numbers represent rf-field amplitude in kHz. The hard pulse durations are shown in µs, while total duration of CP and decoupling are in ms. For ^{1}H→^{13}C transfers, CP with [80%:100%] ramp was applied on the proton channel. For ^{15}N→^{1}H transfers CP with [100%:80%] ramp was applied on the proton channel. For ramped CP, rectanglular brackets indicate the minimal and maximal rf-field of ramped rf-field strengths. The details of ^{1}H→^{13}CA, ^{13}CA→^{15}N and ^{15}N→^{1}H transfers (the shapes, the rf-field strengths and mixing times) are summarized in Tables S7.

Compiling script (Echo/ Anti-Echo mode into STATES mode).

The Echo/Anti-Echo → STATES conversion script was incorporated into a portion of the drift correction script, which was origionally written by Najbauer and Andreas.[21] It handles more complex cases as compared with the default processing, as presented before.[22] The conversion is implemented before the drift correction part. If there is no requirement for drift correction, both ‘the start shift’ and ‘the end shift’ values are set to zero.

For 2D and 3D experiments with a single Echo/Anti-Echo mode for indirect (t_1) dimension, the script faithfully converts the experimental data only for a specific phase cycling setup of a pair of 90°-pulses (inversion real part before element). In the case of 3D experiments with Echo/Anti-

Echo modes for (t_1) and (t_2) indirect dimensions, the first pair of a 90°-pulses should invert the real part and the second should invert the imaginary. The order of fids, when acquired as planes mode should be 321 (numbers indicate the indexing of the dimensions, F1,F2,F3).

3D experiments with double Echo/Anti-Echo modes can also be processed directly in Topspin without the script. In that case, the obtained spectrum should be scaled by a factor of 0.5.

BRUKER PULSE PROGRAMS

The width of SPEPS pulses is automatically calculated using the ‘cnst31’ parameter (the MAS rate in Hz). Note that there is protection against long acquisitions that occur if this parameter is mistakenly set too low. However, still use with precaution.

The SPEPS SHAPE file

```
##TITLE= SPEPS_XY16.evni
##JCAMP-DX= 5.00 Bruker JCAMP library
##DATA TYPE= Shape Data
##ORIGIN= Bruker BioSpin GmbH
##OWNER= <nmrsu>
##DATE= 2015/11/11
##TIME= 12:48:34
##$SHAPE_PARAMETERS= Type: Const; Start Amplitude 100.0 ; End Amplitude 100.0
##MINX= 1.000000E02
##MAXX= 1.000000E02
##MINY= 0.000000E00
##MAXY= 0.000000E00
##$SHAPE_EXMODE= Excitation
##$SHAPE_TOTROT= 1.000000E02
##$SHAPE_TYPE= Excitation
##$SHAPE_USER_DEF=
##$SHAPE_REPHFAC=
##$SHAPE_BWFAC= 1.120000E00
##$SHAPE_BWFAC50=
##$SHAPE_INTEGFAC= 8.000000E-01
##$SHAPE_MODE= 0
##NPOINTS= 16
##XYPOINTS= (XY..XY)
100,  0
100,  90
```

```
100,  0
100,  90
100,  90
100,  0
100,  90
100,  0
100,  180
100,  270
100,  180
100,  270
100,  270
100,  180
100,  270
100,  180
##END=
```

2D (H)NC

```
;2D (h)NC with SPEPS element for N-C transfer

;Avance III+ version
;parameters:
;p1 : 13C 90 pulse @ plw1
;pl1 : 13C power for 90 pulse

;spnam0 : Ramp90.100
;spoffs0 : 0

;p15 : contact time for H->N CP
;pl20 : 15N power for CP
;spnam10 : Ramp100.90
;spoffs10 : 0


;p2 : 1H 90 pulse @ plw2
;pl2 : 1H power for 90 pulse

;l21: SPEPS blocks
;sp5: ~0.25*MAS C for SPEPS
;sp6: ~075*MAS N for SPEPS

;cpdprg4 : Water suppression with cwX_pl12 @ 15kHz
;cpdprg5 : Water suppression with cwY_pl12 @ 15kHz
;cpdprg2 : 1H decoupling (sltppm_40pTr41 for 1Hprot, waltz16_pl12 for 2Hprot)
;cpdprg3 : 15N decoupling (waltz16_pl17)

;pl12 : 1H decoupling)
;pl18 : 1H decoupling for (cw_pl18)
;pl13: water suppression
;pl17: 15N decoupling power (waltz16)
```

```
;pcpd4 : 33.33 (sltppm 15kHz)
;pcpd2 : 25u (waltz16 10 kHz) - 33.33 (sltppm 15kHz)
;pcpd3 : 25u (waltz16 10 kHz)


;$COMMENT=basic cp experiment, arbitrary contact and decoupling schemes
;$CLASS=Solids
;$DIM=1D
;$TYPE=cross polarisation
;$SUBTYPE=simple 1D
;$OWNER=Bruker
prosol relations=<solids_cp>

#include <Avancesolids.incl>

;cnst20 : RF field achieved at pl13
;cnst21 : on resonance, usually = 0
;cnst22 : positive LG offset
;cnst23 : negative LG offset
;cnst24 : additional LG-offset
;cnst11 : to adjust t=0 for acquisition, if digmod = baseopt
"acqt0=1u*cnst11"
"in0=inf1"
"in30=inf1"
"d0=0.0"
"p11 = (16*(2s/cnst31))"

define delay mix
"mix = (l21*(p11))"

1m
  if "p15   > 15m"  goto Problem
  if "aq    > 56m"    goto Problem
  if "mix    > 15m"    goto Problem
  goto PassParams
Problem, 1m
  print " cnst31 is too low; aq or p15 are too long."
  goto HaltAcqu
PassParams, 1m


1 ze
  mix
2 d1 do:f2 do:f3
  (p2  pl2 ph1):f2
  (p15 pl20 ph2):f3 (p15:sp0 ph9):f2

  1u cpds2:f2
if "p1*2 > d0" goto RAWEVOL
   (center (d0) (p1*2 ph0 pl1):f1)
```

```
if "p1*2 <= d0" goto DECOFF

RAWEVOL, 1u
  d0
DECOFF, 1u do:f2

; echo-antiecho determination
(p7 pl7 ph21):f3        ; 1st half of 180
(p7 pl7 ph22):f3        ; 2nd half of 180

3
  (p11:sp6 ph17):f3 (p11:sp5 ph10):f1
  lo to 3 times l21

  1u cpds2:f2
  1u cpds3:f3
  go=2 ph31
  1m do:f2     do:f3
  10m mc #0 to 2
  F1EA(calph(ph22, +180),caldel(d0, +in0))  ;15N

HaltAcqu, 1m      ;jump address for protection files
exit              ;quit

ph0= 0
ph1= 1 3
ph9= 0
ph2=  0 0 0 0 2 2 2 2

ph10= 0 0 1 1
ph17= 0 0 3 3

ph21= 1
ph22= 3

ph31= 1 3 3 1 3 1 1 3
```

2D (H)CAN

```
; 2D (H)CAN with SPEPS for CA-N transfer

;Avance III version
;parameters:
;p2 : 1H 90 pulse duration
;p3 : 13C 90 pulse duration
;p7 : 15N 90 pulse duration
;p30 : water suppression time (30-200 ms)
;cnst21 : CO offset in ppm (173.7)
;cnst22 : CA offset in ppm (53.7)
;cnst23 : CO/CO offset in ppm (113.7)
;d1 : recycle delay
;d0 : C incremental delay (t1)
```

```
;d10 : N incremental delay (t2)
;in10    : 1/2 increment for 15N evolution
;in0   : 1/2 increment for 13CA evolution
;cpdprg1 : tppm (at pl13) or waltz (at pl13)
;cpdprg4 : cwY (at pl12)
;cpdprg5 : cwX (at pl12)
;pcpd1   : pulse length in decoupling sequence (2xtau_r for tppm, 25us for 10kHz waltz)
;pl2    : power level of 1H hard pulse
;pl12   : power level of decoupling (10-15 kHz)
;pl13   : power level for water suppression
;spnam1  : 1H shape for 1H->13CA CP (ramp 10-20%)
;spoal1  : N/A
;spoff1 : [ON/RES]
;sp1    : 1H power level during 1H->13CA CP
;spnam10 : 1H shape for 15N->1H(N) CP (ramp 10-20%)
;spoal10  : N/A
;spoff10 : [ON/RES]
;sp10    : 1H power level during 15N->1H CP
;cpdprg2 : 15N decoupling pattern during acq (waltz-16)
;p17     : contact time 15N->1H(N) CP (300-500 us)
;pcpd2   : pulse length in 15N decoupling sequence (25 us)
;pl7    : power level for 15N hard pulse
;pl16   : power level for 15N decoupling (corr. to 10 kHz)
;pl20   : 15N power level for 15N->1H CP
;spnam2  : 15N shape for 13CA->15N CP (tan-c100-w10pct)
;sp2    : 15N power level for 13CA->15N CP
;spoal2  : N/A
;spoff2 : [ON/RES]
;p15     : contact time 1H->13CA CP (5 ms)
;p16     : contact time 13CA->15N CP (10 ms)
;p18     : Q3 CO pulse duration
;p19     : Q3 CA pulse duration
;pcpd3   : pulse length in 13C decoupling sequence (25 us)
;cpdprg3 : 13CO/CA decoupling pattern during 15N evol (waltz-16)
;pl3    : power level of 13C hard pulse [REFERENCE]
;pl17   : power level for 13CO/CA decoupling (10 kHz)
;spnam9  : 13C shape for 13CA->15N CP (rectangle)
;sp9     : 13C power for 13CA->15N CP
;spoal9  : N/A
;spoff9 : [ON/RES CO]
;spnam18 : 13CO selective pulse shape (Q3)
;spoal18  : N/A
;spoff18 : [ON/RES CO]
;spnam19 : 13CA selective pulse shape (Q3)
;spoal19  : N/A
;spoff19 : [ON/RES CA]
;spnam29 : 13C shape for 1H->13CO CP (rectangle)
;sp29   : 13C power level for 1H->13CO CP
;spoal29  : N/A
;spoff29 : [ON/RES CO]
;zgoptns : -Dfslg, -Dlacq, or blank
```

```
;td1 : number of C increments
;td2 : number of N increments

;l21: SPEPS blocks
;sp5: ~0.25*MAS C for SPEPS
;sp6: ~0.75*MAS N for SPEPS

;$COMMENT=Inverse Cp with INEPT CBCA mixing
;$CLASS=Solids
;$DIM=3D
;$TYPE=H detect
;$SUBTYPE=Heteronuclear
;$OWNER=CRMN

#include <Avancesolids.incl>

; Start evolutions from exactly 0
"d0=0.0"

; 1H settings
"spoal1=0.5"   ; default value (irrelevant)
"spoff1=0.0"    ; on-resonance
"spoal10=0.5"  ; default value (irrelevant)
"spoff10=0.0"   ; on-resonance
"plw16=plw2*(pow(p2/25,2))"     ; 1H waltz 10kHz decoupling power level

; 15N settings
"pcpd2=25"                      ;does not work!
"spoal2=0.5"   ; default value (irrelevant)
"spoff2=0.0"     ; on-resonance

; 13C settings
"plw17=plw3*(pow(p3/25,2))"     ; 13C waltz 10kHz decoupling power level

"cnst22 = (sfo3-bf3)*1000000/bf3"  ; CA frequency offset (ppm)
"cnst21 = cnst22+(173.7-53.7)"     ; CO frequency offset (ppm)
"cnst23 = cnst22+(113.7-53.7)"    ; the offset half-way CO and CA (ppm)

"p18=3.412/(95.0*bf3/1000000)"    ; 95 ppm bandwidth (safe)
"spw18=plw3*pow((0.5/(p18*0.1515))/(0.25/p3),2)" ;  Q3 power level
"spoal18=0.5"                     ; default value (irrelevant)
"spoff18=bf3*((cnst21-cnst22)/1000000)"

"p19=3.412/(105.0*bf3/1000000)"      ; 95 ppm bandwidth (safe)
"spw19=plw3*pow((0.5/(p19*0.1515))/(0.25/p3),2)" ;  Q3 power level
"spoal19=0.5"                          ; default value (irrelevant)
"spoff19=0.0"                      ; CA frequency

"spoal9=0.0"                      ; needed for offset on C
"spoff9=0.0"                      ; on-resonance
```

```
"spoal29=1.0"                    ; needed for offset on C
"spoff29=0.0"                    ; on-resonance CA

"in0=inf1/2"


;cnst11 : to adjust t=0 for acquisition, if digmod = baseopt
"acqt0=1u*cnst11"

"p11 = (16*(2s/cnst31))"

define delay mix
"mix = (l21*p11)"


1m
  if "p15   > 15m"  goto Problem
  if "aq    > 56m"    goto Problem
  if "mix    > 15m"    goto Problem
  goto PassParams
Problem, 1m
  print " cnst31 is too low; aq or p15 are too long."
  goto HaltAcqu
PassParams, 1m

1 ze
  mix
2 d1 do:f2 do:f3
#include <p15_prot.incl>
#include <aq_prot.incl>

  ;1u fq=0:f3
  1u fq=cnst22(bf ppm):f3
  (p2 pl2 ph3):f2

  (p15:sp29 ph16):f3 (p15:sp1 ph15):f2
  1u cpds2:f2
  (p18:sp18 ph2):f3  ;CO selective Pi
  1u
  (p19:sp19 ph2):f3   ;CA selective Pi
  1u
  d0
  (center (p7*2 ph0 pl7):f1 (p18:sp18 ph2):f3)  ;CO selective Pi
  d0
  1u do:f2

; echo-antiecho determination
(p3 pl3 ph4):f3        ; 1st half of 180
(p3 pl3 ph25):f3        ; 2nd half of 180

3
```

```
  (p11:sp5 ph10):f3 (p11:sp6 ph11):f1
  lo to 3 times l21
  1u fq=cnst23(bf ppm):f3
  1u cpds2:f2  cpds3:f3
  go=2 ph31
  1m do:f2 do:f3

  10m mc #0 to 2

F1EA(calph(ph25, +180),caldel(d0, +in0))  ;13C


HaltAcqu, 1m      ;jump address for protection files
exit                    ;quit

ph0  = 0
ph1  = 1
ph3  = 1 3
ph15 = 0
ph16= 0 0 0 0 2 2 2 2
ph2 = 0
ph4= 1
ph25= 3
ph10= 0 0 3 3
ph11= 0 0 1 1

ph31 = 1 3 3 1 3 1 1 3
```

3D (H)CANH

```
; 3D (H)CANH with SPEPS element for 13C->15N transfer
; Developed at CRMN in the group of G. Pintacuda and modified at MPI-NAT group of L. Andreas

;Avance III version
;parameters:
;p1 : 1H 90 pulse duration
;p3 : 13C 90 pulse duration
;p7 : 15N 90 pulse duration
;p30 : water suppression time (30-200 ms)
;cnst21 : CO offset in ppm (173.7)
;cnst22 : CA offset in ppm (53.7)
;cnst23 : CO/CO offset in ppm (113.7)
;d1 : recycle delay
;d0 : C incremental delay (t1)
;d10 : N incremental delay (t2)
;in10    : 1/2 increment for 15N evolution
;in0   : 1/2 increment for 13CA evolution
;cpdprg1 : tppm (at pl13) or waltz (at pl13)
;cpdprg4 : cwY (at pl12)
;cpdprg5 : cwX (at pl12)
```

;pcpd1 : pulse length in decoupling sequence (2xtau_r for tppm, 25us for 10kHz waltz)
;pl1 : power level of 1H hard pulse
;pl12 : power level of decoupling (10-15 kHz)
;pl13 : power level for water suppression
;spnam1 : 1H shape for 1H->13CA CP (ramp 10-20%)
;spoal1 : N/A
;spoff1 : [ON/RES]
;sp1 : 1H power level during 1H->13CA CP
;spnam10 : 1H shape for 15N->1H(N) CP (ramp 10-20%)
;spoal10 : N/A
;spoff10 : [ON/RES]
;sp10 : 1H power level during 15N->1H CP
;cpdprg2 : 15N decoupling pattern during acq (waltz-16)
;p17 : contact time 15N->1H(N) CP (300-500 us)
;pcpd2 : pulse length in 15N decoupling sequence (25 us)
;pl7 : power level for 15N hard pulse
;pl16 : power level for 15N decoupling (corr. to 10 kHz)
;pl2 : power level for 15N hard pulse
;pl20 : 15N power level for 15N->1H CP
;spnam2 : 15N shape for 13CA->15N CP (tan-c100-w10pct)
;sp2 : 15N power level for 13CA->15N CP
;spoal2 : N/A
;spoff2 : [ON/RES]
;p15 : contact time 1H->13CA CP (5 ms)
;p16 : contact time 13CA->15N CP (10 ms)
;p18 : Q3 CO pulse duration
;p19 : Q3 CA pulse duration
;pcpd3 : pulse length in 13C decoupling sequence (25 us)
;cpdprg3 : 13CO/CA decoupling pattern during 15N evol (waltz-16)
;pl3 : power level of 13C hard pulse [REFERENCE]
;pl17 : power level for 13CO/CA decoupling (10 kHz)
;spnam9 : 13C shape for 13CA->15N CP (rectangle)
;sp9 : 13C power for 13CA->15N CP
;spoal9 : N/A
;spoff9 : [ON/RES CO]
;spnam18 : 13CO selective pulse shape (Q3)
;spoal18 : N/A
;spoff18 : [ON/RES CO]
;spnam19 : 13CA selective pulse shape (Q3)
;spoal19 : N/A
;spoff19 : [ON/RES CA]
;spnam29 : 13C shape for 1H->13CO CP (rectangle)
;sp29 : 13C power level for 1H->13CO CP
;spoal29 : N/A
;spoff29 : [ON/RES CO]
;zgoptns : -Dfslg, -Dlacq, or blank
;td1 : number of C increments
;td2 : number of N increments

;l21: Number SPEPS elements
;sp5: ~0.25*MAS C for SPEPS

```
;sp6: ~0.75*MAS N for SPEPS

;$COMMENT=Inverse Cp with INEPT CBCA mixing
;$CLASS=Solids
;$DIM=3D
;$TYPE=H detect
;$SUBTYPE=Heteronuclear
;$OWNER=CRMN

#include <Avancesolids.incl>

; Start evolutions from exactly 0
"d10=0.0"
"d0=0.0"

; 1H settings
"spoal1=0.5"   ; default value (irrelevant)
"spoff1=0.0"    ; on-resonance
"spoal10=0.5"  ; default value (irrelevant)
"spoff10=0.0"   ; on-resonance

; 15N settings
"pcpd2=25"                        ;does not work!
"plw2=plw7"
"plw16=plw2*(pow(p7/25,2))"     ; 15N waltz 10kHz decoupling power level
"spoal2=0.5"   ; default value (irrelevant)
"spoff2=0.0"     ; on-resonance

; 13C settings
"plw17=plw3*(pow(p3/25,2))"     ; 13C waltz 10kHz decoupling power level

"cnst22 = (sfo3-bf3)*1000000/bf3"  ; CA frequency offset (ppm)
"cnst21 = cnst22+(173.7-53.7)"     ; CO frequency offset (ppm)
"cnst23 = cnst22+(113.7-53.7)"    ; the offset half-way CO and CA (ppm)

"p18=3.412/(95.0*bf3/1000000)"    ; 95 ppm bandwidth (safe)
"spw18=plw3*pow((0.5/(p18*0.1515))/(0.25/p3),2)" ;  Q3 power level
"spoal18=0.5"                      ; default value (irrelevant)
"spoff18=bf3*((cnst21-cnst22)/1000000)"

"p19=3.412/(105.0*bf3/1000000)"      ; 95 ppm bandwidth (safe)
"spw19=plw3*pow((0.5/(p19*0.1515))/(0.25/p3),2)" ;  Q3 power level
"spoal19=0.5"                            ; default value (irrelevant)
"spoff19=0.0"                      ; CA frequency

"spoal9=0.0"                       ; needed for offset on C
"spoff9=0.0"                       ; on-resonance

"spoal29=1.0"                      ; needed for offset on C
"spoff29=0.0"                      ; on-resonance CA
```

```
"in0=inf1/2"
"in10=inf2/2"


;"acqt0=0"                ; baseopt correction

"p10 = (16*(2s/cnst31))"

define delay mix
"mix = (l21*p10)"

1m
  if "p15   > 15m"  goto Problem
  if "aq    > 56m"    goto Problem
  if "p17    > 7m"    goto Problem
  if "mix    > 15m"    goto Problem
  goto PassParams
Problem, 1m
  print " cnst31 is too low; aq, p15 or p17 are too long."
  goto HaltAcqu
PassParams, 1m

1 ze
  mix
2 d1 do:f2
#include <p15_prot.incl>
#include <aq_prot.incl>

  ;1u fq=0:f3
  1u fq=cnst22(bf ppm):f3
  (p1 pl1 ph3):f1

  (p15:sp29 ph15):f3 (p15:sp1 ph16):f1

  ;(p3 pl8 ph4):f3 ; 1st half of 180

  1u cpds1:f1
  (p18:sp18 ph2):f3  ;CO selective Pi
  1u
  (p19:sp19 ph2):f3   ;CA selective Pi
  1u
  d0
  (center (p7*2 ph0 pl7):f2 (p18:sp18 ph2):f3)  ;CO selective Pi
  d0
  1u do:f1

; echo-antiecho determination
(p3 pl3 ph4):f3 ; 1st half of 180
(p3 pl3 ph25):f3 ; 2nd half of 180
```

```
3
  (p10:sp5 ph10):f3 (p10:sp6 ph12):f2
  lo to 3 times l21
  1u fq=cnst23(bf ppm):f3
  1u cpds1:f1
  d10
  (p3*2 pl3 ph0):f3
  d10
  1u do:f1
  (p7 pl7 ph5):f2
 ;water suppression
  (p30*0.25 pl13 ph0):f1
  (p30*0.25 pl13 ph1):f1
  (p30*0.25 pl13 ph0):f1
  (p30*0.25 pl13 ph1):f1
 ;water suppression
  (p7 pl7 ph6):f2

  (p17 pl20 ph7):f2 (p17:sp10 ph17):f1

  1u cpds2:f2
  go=2 ph31
  1m do:f2

  10m mc #0 to 2

 F1EA(calph(ph25, +180),caldel(d0, +in0))  ;13C
 F2PH(calph(ph5, +90), caldel(d10, +in10)) ;15N



HaltAcqu, 1m      ;jump address for protection files
exit                ;quit

ph0  = 0
ph1  = 1
ph3  = 1
ph15 = 2
ph16 = 0 0 2 2
ph5 =  1
ph6 =  3
ph2 = 0
ph4= 1
ph25= 3
ph10= 0 3
ph12= 0 1
ph21= 0
ph22= 2

ph7  = 2 2 2 2 0 0 0 0
ph17 = 0
```

ph31 = 1 3 3 1 3 1 1 3

3D (H)CANCO

```
;3D (H)CANCO with SPEPS elements for CA->N and N->CO transfers
;developed at MPI-NAT. Group of L. Andreas


;Avance III version
;parameters:
;p2 : 1H 90 pulse duration
;p3 : 13C 90 pulse duration
;p7 : 15N 90 pulse duration
;p30 : water suppression time (30-200 ms)
;cnst21 : CO offset in ppm (173.7)
;cnst22 : CA offset in ppm (53.7)
;cnst23 : CO/CO offset in ppm (113.7)
;d1 : recycle delay
;d0 : C incremental delay (t1)
;d10 : N incremental delay (t2)
;in10    : 1/2 increment for 15N evolution
;in0   : 1/2 increment for 13CA evolution
;cpdprg1 : tppm (at pl13) or waltz (at pl13)
;cpdprg4 : cwY (at pl12)
;cpdprg5 : cwX (at pl12)
;pcpd1   : pulse length in decoupling sequence (2xtau_r for tppm, 25us for 10kHz waltz)
;pl2    : power level of 1H hard pulse
;pl12   : power level of decoupling (10-15 kHz)
;pl13   : power level for water suppression
;spnam1  : 1H shape for 1H->13CA CP (ramp 10-20%)
;spoal1  : N/A
;spoff1 : [ON/RES]
;sp1    : 1H power level during 1H->13CA CP
;spnam10 : 1H shape for 15N->1H(N) CP (ramp 10-20%)
;spoal10  : N/A
;spoff10 : [ON/RES]
;sp10    : 1H power level during 15N->1H CP
;cpdprg2 : 15N decoupling pattern during acq (waltz-16)
;p17     : contact time 15N->1H(N) CP (300-500 us)
;pcpd2   : pulse length in 15N decoupling sequence (25 us)
;pl7    : power level for 15N hard pulse
;pl16   : power level for 15N decoupling (corr. to 10 kHz)
;pl20   : 15N power level for 15N->1H CP
;spnam2  : 15N shape for 13CA->15N CP (tan-c100-w10pct)
;sp2    : 15N power level for 13CA->15N CP
;spoal2  : N/A
;spoff2 : [ON/RES]
;p15     : contact time 1H->13CA CP (5 ms)
;p16     : contact time 13CA->15N CP (10 ms)
;p18     : Q3 CO pulse duration
```

```
;p19     : Q3 CA pulse duration
;pcpd3   : pulse length in 13C decoupling sequence (25 us)
;cpdprg3 : 13CO/CA decoupling pattern during 15N evol (waltz-16)
;pl3    : power level of 13C hard pulse [REFERENCE]
;pl17   : power level for 13CO/CA decoupling (10 kHz)
;spnam9  : 13C shape for 13CA->15N CP (rectangle)
;sp9     : 13C power for 13CA->15N CP
;spoal9  : N/A
;spoff9 : [ON/RES CO]
;spnam18 : 13CO selective pulse shape (Q3)
;spoal18  : N/A
;spoff18 : [ON/RES CO]
;spnam19 : 13CA selective pulse shape (Q3)
;spoal19  : N/A
;spoff19 : [ON/RES CA]
;spnam29 : 13C shape for 1H->13CA CP (rectangle)
;sp29   : 13C power level for 1H->13CA CP
;spoal29  : N/A
;spoff29 : [ON/RES CO]
;zgoptns : -Dfslg, -Dlacq, or blank
;td1 : number of C increments
;td2 : number of N increments

;l21: CAN blocks
;l22: NCO blocks
;sp5: ~0.25*MAS C for SPEPS
;sp6: ~0.75*MAS N for SPEPS

;$COMMENT=Inverse Cp with INEPT CBCA mixing
;$CLASS=Solids
;$DIM=3D
;$TYPE=H detect
;$SUBTYPE=Heteronuclear
;$OWNER=CRMN

#include <Avancesolids.incl>

; Start evolutions from exactly 0
"d0=0.0"
"d10=0.0"

; 1H settings
"spoal1=0.5"   ; default value (irrelevant)
"spoff1=0.0"    ; on-resonance
"spoal10=0.5"  ; default value (irrelevant)
"spoff10=0.0"   ; on-resonance
"plw16=plw2*(pow(p2/25,2))"     ; 1H waltz 10kHz decoupling power level

; 15N settings
"pcpd2=25"                      ;does not work!
"spoal2=0.5"   ; default value (irrelevant)
```

```
"spoff2=0.0"     ; on-resonance
"plw17=plw7*(pow(p7/25,2))"     ; 15N waltz 10kHz decoupling power level

; 13C settings
"cnst21 = (sfo1-bf1)*1000000/bf1"  ; CO frequency offset (ppm)
"cnst22 = cnst21-(173.7-54)"     ; CA frequency offset (ppm)
"cnst23 = cnst21-(113.7-54)"    ; the offset half-way CO and CA (ppm)

"p18=3.412/(90.0*bf1/1000000)"    ; 95 ppm bandwidth (safe)
"spw18=plw1*pow((0.5/(p18*0.1515))/(0.25/p1),2)" ;  Q3 power level
"spoal18=0.5"                      ; default value (irrelevant)
"spoff18=bf1*((cnst21-cnst22)/1000000)"

"p19=3.412/(90.0*bf1/1000000)"      ; 95 ppm bandwidth (safe)
"spw19=plw1*pow((0.5/(p19*0.1515))/(0.25/p1),2)" ;  Q3 power level
"spoal19=0.5"                           ; default value (irrelevant)
"spoff19=0.0"   ; CA frequency

"spoal9=0.0"                       ; needed for offset on C
"spoff9=0.0"                       ; on-resonance

"spoal29=1.0"                       ; needed for offset on C
"spoff29=0.0"                       ; on-resonance CA

"in0=inf1/2"
"in10=inf2/2"


;cnst11 : to adjust t=0 for acquisition, if digmod = baseopt
"acqt0=1u*cnst11"

"p10 = (16*(2s/cnst31))"

define delay mix
"mix = (l21*(p10))"

define delay mix2
"mix2 = (l22*(p10))"

1m
  if "p15   > 15m"  goto Problem
  if "aq    > 56m"    goto Problem
  if "mix    > 15m"    goto Problem
  if "mix2    > 15m"    goto Problem
  goto PassParams
Problem, 1m
  print "cnst31 is too low; aq or p15 are too long."
  goto HaltAcqu
PassParams, 1m

1 ze
```

```
  mix
  mix2
2 d1 do:f2 do:f3
#include <p15_prot.incl>
#include <aq_prot.incl>

  1u fq=cnst22(bf ppm):f1
  (p2 pl2 ph3):f2

  (p15:sp29 ph15):f1 (p15:sp1 ph16):f2
  1u  cpds2:f2
  (p18:sp18 ph2):f1  ;CO selective Pi
  1u
  (p19:sp19 ph2):f1   ;CA selective Pi
  1u
  d0
  (center (p7*2 ph0 pl7):f3 (p18:sp18 ph2):f1)  ;CO selective Pi
  d0
  1u do:f2
; echo-antiecho determination
  (p1 pl1 ph4):f1   ; 1st half of 180

  (p1 pl1 ph14):f1 ; 2nd half of 180

  ;(p1 pl1 ph1):f1

3
  (p10:sp5 ph10):f1 (p10:sp6 ph11):f3
  lo to 3 times l21
  1u cpds2:f2 cpds1:f1
  d10
  d10
  1u do:f2 do:f1
  1u fq=cnst21(bf ppm):f1 ;move offset from CA to CO

; echo-antiecho determination
  (p7 pl7 ph24):f3 ; 1st half of 180
  (p7 pl7 ph25):f3 ; 2nd half of 180

4
  (p10:sp5 ph20):f1 (p10:sp6 ph21):f3
  lo to 4 times l22
  1u cpds2:f2 cpds3:f3
 ; (p1 pl1 ph1):f1
  go=2 ph31
  1m do:f2 do:f3

  10m mc #0 to 2

F1EA(calph(ph14, +180),caldel(d0, +in0))  ;13C
F2EA(calph(ph25, +180) & calph(ph14, +180),caldel(d10, +in10))  ;15N
```

```
HaltAcqu, 1m      ;jump address for protection files
exit                ;quit

ph0  = 0
ph1  = 1
ph2 = 0

ph3  = 1 3
ph15 = 0
ph16 = 0

ph4= 1
ph14= 3

ph10= 0 0 3 3
ph11= 0 0 1 1

ph24= 0
ph25= 2

ph20= 1 0 1 0
ph21= 1 0 1 0 3 2 3 2

ph31 = 1 3 3 1 3 1 1 3
```

REFERENCES

1. Hartmann, S. R. & Hahn, E. L. Nuclear Double Resonance in the Rotating Frame. *Phys. Rev.* **128**, 2042–2053 (1962).
2. Nimerovsky, E., Becker, S. & Andreas, L. B. Windowed cross polarization at 55 kHz magic-angle spinning. *J. Magn. Reson.* **349**, 107404 (2023).
3. Emshwiller, M., Hahn, E. L. & Kaplan, D. Pulsed Nuclear Resonance Spectroscopy. *Phys. Rev.* **118**, 414–424 (1960).
4. Gullion, T., Baker, D. B. & Conradi, M. S. New, compensated Carr-Purcell sequences. *J. Magn. Reson. 1969* **89**, 479–484 (1990).
5. Xue, K., Nimerovsky, E., Tekwani Movellan, K. A., Becker, S. & Andreas, L. B. Backbone Torsion Angle Determination Using Proton Detected Magic-Angle Spinning Nuclear Magnetic Resonance. *J. Phys. Chem. Lett.* **13**, 18–24 (2022).

6. Paulson, E. K., Martin, R. W. & Zilm, K. W. Cross polarization, radio frequency field homogeneity, and circuit balancing in high field solid state NMR probes. *J. Magn. Reson.* **171**, 314–323 (2004).
7. Engelke, F. Electromagnetic wave compression and radio frequency homogeneity in NMR solenoidal coils: Computational approach. *Concepts Magn. Reson.* **15**, 129–155 (2002).
8. Gupta, R., Hou, G., Polenova, T. & Vega, A. J. RF INHOMOGENEITY AND HOW IT CONTROLS CPMAS. *Solid State Nucl. Magn. Reson.* **72**, 17–26 (2015).
9. Blahut, J., Brandl, M. J., Pradhan, T., Reif, B. & Tošner, Z. Sensitivity-Enhanced Multidimensional Solid-State NMR Spectroscopy by Optimal-Control-Based Transverse Mixing Sequences. *J. Am. Chem. Soc.* (2022) doi:10.1021/jacs.2c06568.
10. Nimerovsky, E. & Goldbourt, A. Insights into the spin dynamics of a large anisotropy spin subjected to long-pulse irradiation under a modified REDOR experiment. *J. Magn. Reson.* **225**, 130–141 (2012).
11. Schnell, J. R. & Chou, J. J. Structure and mechanism of the M2 proton channel of influenza A virus. *Nature* **451**, 591–595 (2008).
12. Andreas, L. B., Eddy, M. T., Pielak, R. M., Chou, J. & Griffin, R. G. Magic Angle Spinning NMR Investigation of Influenza A M218−60: Support for an Allosteric Mechanism of Inhibition. *J. Am. Chem. Soc.* **132**, 10958–10960 (2010).
13. Hoyer, W. *et al.* Dependence of α-Synuclein Aggregate Morphology on Solution Conditions. *J. Mol. Biol.* **322**, 383–393 (2002).
14. Thakur, R. S., Kurur, N. D. & Madhu, P. K. Swept-frequency two-pulse phase modulation for heteronuclear dipolar decoupling in solid-state NMR. *Chem. Phys. Lett.* **426**, 459–463 (2006).

15. Li, Y., Wylie, B. J. & Rienstra, C. M. Selective refocusing pulses in magic-angle spinning NMR: Characterization and applications to multi-dimensional protein spectroscopy. *J. Magn. Reson.* **179**, 206–216 (2006).

16. Hediger, S., Meier, B. H. & Ernst, R. R. Adiabatic passage Hartmann-Hahn cross polarization in NMR under magic angle sample spinning. *Chem. Phys. Lett.* **240**, 449–456 (1995).

17. Shaka, A. J., Keeler, J., Frenkiel, T. & Freeman, R. An improved sequence for broadband decoupling: WALTZ-16. *J. Magn. Reson. 1969* **52**, 335–338 (1983).

18. Zhou, D. H. & Rienstra, C. M. High-performance solvent suppression for proton detected solid-state NMR. *J. Magn. Reson.* **192**, 167–172 (2008).

19. Baldus, M., Petkova, A. T., Herzfeld, J. & Griffin, R. G. Cross polarization in the tilted frame: assignment and spectral simplification in heteronuclear spin systems. *Mol. Phys.* **95**, 1197–1207 (1998).

20. Laage, S. *et al.* Band-Selective 1H−13C Cross-Polarization in Fast Magic Angle Spinning Solid-State NMR Spectroscopy. *J. Am. Chem. Soc.* **130**, 17216–17217 (2008).

21. Najbauer, E. E. & Andreas, L. B. Correcting for magnetic field drift in magic-angle spinning NMR datasets. *J. Magn. Reson.* **305**, 1–4 (2019).

22. Wong, L. E., Maier, J., Wienands, J., Becker, S. & Griesinger, C. Sensitivity-Enhanced Four-Dimensional Amide–Amide Correlation NMR Experiments for Sequential Assignment of Proline-Rich Disordered Proteins. *J. Am. Chem. Soc.* **140**, 3518–3522 (2018).
